# Supplementary material for: General cognitive function and the brain’s structural connectome
Source: bioRxiv. 2026 Apr 1:2026.03.30.715223. Preprint. [Version 1] doi: 10.64898/2026.03.30.715223 (PMC13060379; doi:10.64898/2026.03.30.715223)
Supplement: Supplement 1 [file media-1.pdf]

Supplementary material - General cognitive function and the brain's structural connectome

Colin R. Buchanan[1,2,3] *, Joanna E. Moodie[1,2], Venia Batziou[4], Eleanor L. S. Conole[1,2,5], Susana Muñoz Maniega[1,3,6], Mathew A. Harris[1,2], Hon Wah Yeung[1,2], Janie Corley[1,2], David C. Liewald[1,2], Paul Redmond[1,2], J. Douglas Steele[7], Gordon D. Waiter[3,8], Heather C. Whalley[9,10], Andrew M. McIntosh[9,10], Joanna M. Wardlaw[1,3,6,11], Mark E. Bastin[1,3,6], Ian J. Deary[1,2], Elliot M. Tucker-Drob[12], Simon R. Cox[1,2,3]

1. Lothian Birth Cohorts, Department of Psychology, The University of Edinburgh, Edinburgh, UK.
2. Lothian Birth Cohorts, Edinburgh Futures Institute, The University of Edinburgh, Edinburgh, UK.
3. Scottish Imaging Network, A Platform for Scientific Excellence (SINAPSE) Collaboration, UK.
4. College of Medicine & Veterinary Medicine, The University of Edinburgh, Edinburgh, UK.
5. Department of Biochemistry, University of Oxford, Oxford, UK.
6. Centre for Clinical Brain Sciences, The University of Edinburgh, Edinburgh, UK.
7. Division of Neuroscience, University of Dundee, Dundee, UK
8. Aberdeen Biomedical Imaging Centre, University of Aberdeen, Aberdeen UK
9. Department of Psychiatry, The University of Edinburgh, Edinburgh, UK.
10. Generation Scotland, Centre for Medical Informatics, Usher Institute, The University of Edinburgh, Edinburgh, UK
11. UK Dementia Research Institute Centre, The University of Edinburgh, Edinburgh, UK
12. Department of Psychology, The University of Texas at Austin, Austin, USA

Detailed materials and methods

Participants

We evaluated the associations between white matter networks and general cognitive function (*g*) using data from three large and well-characterised cohorts. The largest cohort was UK Biobank (UKB; *n* = 37,284; aged 45–82 years)[1], which was randomly split into two halves, comprising a main sample (*n* = 18,642), and a held-out sample for replication purposes. The other cohorts were Generation Scotland: Stratifying Resilience and Depression Longitudinally (STRADL; *n* = 937; 26–84 years)[2], and the Lothian Birth Cohort 1936 (LBC1936; *n* = 603; ~73 years)[3,4]. This resulted in a total sample size of 20,182 participants for meta-analysis. All participants provided informed consent to participate as stated below.

The UK Biobank is a large-scale epidemiology study which recruited approximately 500,000 community-dwelling adults aged 40–69 years from across Great Britain between 2006 and 2010[1]. Participants provided comprehensive demographic, psychosocial, medical information, blood samples for DNA extraction, physical fitness testing, and cognitive assessment during an initial visit to a UK Biobank assessment centre. A subset (~40,000) underwent brain MRI 9.0 ± 1.8 years after baseline assessment[5]. Participants were generally healthy, and the full connectome sample included 37,284 participants. A subset of 2310 participants (6.2%) had at least one of 26 self-reported neurological conditions (e.g., stroke, epilepsy, multiple sclerosis, meningitis, Parkinson's; see full details below), and these participants were excluded in a supplementary analysis (below). UK Biobank received ethical approval from the North West Multi-centre Research Ethics Committee (REC reference 11/NW/0382). The current study was conducted under approved UK Biobank application number 10279.

STRADL is a population-based study, developed from the Generation Scotland: Scottish Family Health Study (GS:SFHS)[6]. A subset of these participants (*n* = 1188) were invited to take part in an additional study (STRADL), which was primarily designed to study major depressive disorder, although participants were not selected based on depression[2]. STRADL participants underwent brain MRI and data collection included socio-economic and lifestyle variables, physical measures, mental health questionnaires, laboratory samples, and cognitive assessment. Participants were generally healthy, although approximately 30% had a lifetime history of a mood disorder. NHS records were previously obtained as part of the GS:SFHS (05/S1401/89) and subsequent procedures were conducted following an independent, but linked, NHS Tayside Committee on Research Ethics application (14/SS/0039).

The LBC1936 is a longitudinal study of community-dwelling older adults, most of whom took part in the Scottish Mental Survey of 1947 at ~11 years old, and who volunteered to participate in this cohort study at ~70 years old[3,4,7]. Data have been gathered on the influences on cognitive ageing from age 11 into the 8th and 9th decades of life. Participants underwent a thorough assessment including medical interviews, physical fitness tests, extensive cognitive assessments, completion of psycho-social questionnaires, and blood samples for DNA extraction and biomarker analyses. LBC1936 data were drawn from all available participants from the first brain imaging wave (~73 years of age). Participants were generally healthy, with no self-reported symptoms of dementia, while 76 (12.6%) met criteria for neuroradiologically identified stroke. The LBC1936 study was given ethical approval by the Multi-Centre Research Ethics Committee for Scotland, (MREC/01/0/56), the Lothian Research Ethics Committee (LREC/2003/2/29) and the Scotland A Research Ethics Committee (07/MRE00/58).

MRI acquisition

Details of the UKB MRI protocol and processing are freely available[5,8]. All imaging data used in this study were acquired using one of four Siemens Skyra 3T scanners across four UK imaging centres (Cheadle, Newcastle upon Tyne, Reading, and Bristol). 3D T1-weighted volumes were acquired using a magnetization-prepared rapid gradient-echo sequence at 1 × 1 × 1 mm resolution with 208 × 256 × 256 field of view. The dMRI data were acquired using a spin-echo echo-planar imaging sequence (50 b = 1000 s/mm2, 50 b = 2000 s/mm2 and 5 $b \approx 0$ s/mm2) resulting in 100 distinct diffusion-encoding directions. The field of view was 104 × 104 mm with imaging matrix 52 × 52 and 72 slices with slice thickness of 2 mm resulting in 2 × 2 × 2 mm voxels.

Details of the STRADL protocol have been described previously[2]. Acquisition took place at two sites with the MRI acquisition parameters matched as closely as practical: Aberdeen (n = 582, 49%) and Dundee (n = 606, 51%). 3D T1-weighted fast gradient echo volumes with magnetisation preparation were acquired. The scanner used in Aberdeen was a 3T Philips Achieva TX-series MRI system (Philips Healthcare, Best, Netherlands) with a 32-channel phased-array head coil and a back facing mirror (gradients with maximum amplitude 80 mT/m and maximum slew rate 100 T/m/s). For T1-weighted images, 160 sagittal slices were acquired with a field of view of 240 mm and a matrix size of 240 x 240 pixels, giving a resolution of 1 × 1 × 1 mm. For the dMRI protocol, echo-planar (EP) diffusion-weighted whole-brain volumes (b = 1200 s mm^{-2}) were acquired in 64 non-collinear directions, along with eight T2-weighted volumes (b = 0 s mm^{-2}). Sixty contiguous 2.3 mm thick axial slices were acquired with a matrix size of 96 × 94 resulting in 2.3 mm^3 isotropic voxels. The scanner used in Dundee was a Siemens 3T Prisma-FIT (Siemens, Erlangen, Germany) with 20 channel head and neck phased array coil and a back facing mirror (Syngo E11, gradient with max amplitude 80 mT/m and maximum slew rate 200 T/m/s). For T1-weighted images 208 sagittal slices were acquired with a field of view of 256 mm and matrix size 256 × 256 pixels giving a resolution of 1 × 1 × 1 mm. For the dMRI protocol, echo-planar (EP) diffusion-weighted whole-brain volumes (b = 1200 s mm^{-2}) were acquired in 64 non-collinear directions, along with eight T2-weighted volumes (b = 0 s mm^{-2}). Sixty contiguous 2.3 mm thick axial slices were acquired with a matrix size of 96 × 94 resulting in 2.3 mm^3 isotropic voxels.

Details of the LBC1936 MRI protocol have been described previously[9]. All participants underwent brain MRI on the same 1.5T GE Signa Horizon HDx clinical scanner (General Electric, Milwaukee, WI, USA) with a manufacturer supplied 8-channel phased-array head coil. High resolution 3D T1-weighted inversion-recovery prepared, fast spoiled gradient-echo volumes were acquired in the coronal plane with 180 contiguous 1.3 mm thick slices resulting in voxel dimensions of 1 × 1 × 1.3 mm. For the dMRI protocol, single-shot spin-echo echo-planar (EP) diffusion-weighted whole-brain volumes (b = 1000 s mm^{-2}) were acquired in 64 non-collinear directions, along with seven T2-weighted volumes (b = 0 s mm^{-2}). Seventy-two contiguous axial 2 mm thick slices were acquired resulting in 2 mm^3 isotropic voxels.

T1-weighted processing

Each T1-weighted image was segmented into 85 distinct neuroanatomical regions-of-interest (ROIs) using the volumetric segmentation and cortical reconstruction of the FreeSurfer image analysis suite (http://surfer.nmr.mgh.harvard.edu). The Desikan-Killiany atlas was used to delineate 34 cortical structures per hemisphere[10,11]. Subcortical segmentation was applied to obtain the brain stem and eight grey matter structures per hemisphere: accumbens area, amygdala, caudate, hippocampus, pallidum, putamen, thalamus and ventral diencapahlon[12,13]. Grey and white tissue matter masks were also obtained. Cortical processing was conducted locally using FreeSurfer v5.1.0 for LBC1936, and v5.3.0 for STRADL. UKB processing was conducted by the UKB imaging team using FreeSurfer v6.0.

FreeSurfer quality control procedure

The quality control (QC) approach differed by cohort. The Lothian Birth Cohort 1936 (LBC1936) Wave 2 data were collected between 2007-2010 and were processed, edited and underwent QC using FreeSurfer v5.1.0. STRADL data was processed using FreeSurfer v5.3.0. Manual QC of FreeSurfer data for STRADL and LBC1936 involved visual inspection of skull stripping, tissue segmentation and regional boundaries followed by manual editing to rectify segmentation and parcellation errors; participants with uncorrectable errors were excluded. UK Biobank (UKB) segmentations underwent automated quality checking and exclusion, which was conducted by the UKB imaging team[14].

Diffusion MRI processing and tractography

All dMRI data were processed with the FSL toolkit (FMRIB, Oxford University: http://www.fmrib.ox.ac.uk)[15]. However, processing differed between cohorts due to limitations of the acquisition (e.g., absence of reverse phase encodings) and the FSL version available at the time of processing. All LBC1936 (FSL v4.1.9) and STRADL (FSL v5.0.9) data were processed locally as follows. Data underwent brain extraction[16] performed on the T2-weighted EP volumes acquired along with the dMRI data. The brain mask was applied to all volumes after correcting for systematic eddy-current induced imaging distortions and head motion using affine registration to the first T2-weighted EP volume of each participant with 'eddy_correct'[17]. UKB processing was performed by the UKB imaging team (FSL v6.0) using an UKB-specific pipeline that exploits the reverse phase encoding and multi-shell acquisition of the UKB dMRI data[8]. Briefly, UKB processing involved field map generation and subsequent correction for eddy currents, systematic distortions and head motion. For all dMRI volumes, diffusion tensors were fitted at each voxel (FSL dtifit). Water diffusion measures were estimated for: mean diffusivity (MD), which measure magnitudes of molecular water diffusion; and fractional anisotropy (FA), which measures the degree of anisotropic diffusion per voxel[18]. All diffusion measures and tractography were computed in diffusion space.

Whole-brain tractography was performed using an established probabilistic algorithm (BEDPOSTX/ProbtrackX)[19,20]. Probability density functions, which describe the uncertainty in the principal directions of diffusion, were computed with a multi-fibre model (two-fibres for LBC1936 and STRADL; three-fibres for UKB) per voxel[20,21]. Streamlines were then constructed by sampling from these distributions during tracking using 100 Markov Chain Monte Carlo iterations with a fixed step size of 0.5 mm between successive points. Tracking was initiated from all white matter voxels using stopping criteria as described previously[22].

Network construction

Construction of whole-brain networks was harmonised as much as practical across cohorts using an automated connectivity mapping pipeline. For all participants with compatible T1 and dMRI data, white matter structural networks were constructed using the 85 neuroanatomical regions described above and the set of streamlines generated by tractography resulting in 85 × 85 connectivity matrices[22]. Networks were constructed by identifying connections between all ROI pairs. The endpoint of a streamline was recorded as the first ROI encountered (if any) when tracking from the seed location.

For each participant, three network types were computed reflecting different aspects of connectivity. Streamline count (SC) was computed by recording the total streamline count (uncorrected) between each pair of ROIs. FA and MD networks were computed by computing the mean value of each measure in all voxels along the interconnecting streamlines between each pair of ROIs. In addition, a variant of streamline count termed streamline density (SD) was computed by dividing counts by the volumes of the grey matter ROIs involved[23].
As network density, i.e., the number of network connections identified, is known to depend on MRI field strength, acquisition parameters and dMRI processing, we chose to apply a cross-cohort

network thresholding procedure to match network density across cohorts. First, consistency-thresholding[24] was independently applied to each cohort to remove putatively spurious connections at a threshold level retaining the top 30% most consistent connections – this density was previously determined from a large single-scanner study in a portion of the UKB data[22]. Second, the three cohort masks (containing non-identical sets of connections) were combined and only connections (edges) found to be present in all three cohorts were retained. The resulting cross-cohort mask was then applied to each network across all participants.

For each network, three global graph-theoretic metrics were computed using weighted metrics from the Brain Connectivity Toolbox[25]: mean edge weight (mean of all edge weights per participant), global network efficiency (a measure of integration) and network clustering coefficient (a measure of segregation). Global efficiency measures how easily information can flow across an entire network, reflecting the network's overall ability to connect different nodes efficiently. Greater global efficiency signifies more effective 'wiring' across the entire brain. The mean clustering coefficient measures on average how many local clusters exist around a node in terms of its direct neighbours, indicating how tightly interconnected neighbouring nodes are within a network. A higher nodal coefficient indicates that nearby network nodes are densely interconnected, potentially reflecting specialised functions within those clusters. Although global graph-theory metrics are known to be highly correlated[26], we chose to assess these three commonly used metrics. At the node level, only weighted local efficiency was assessed, quantifying how efficiently information is exchanged within the immediate neighbourhood of each node based on the strength of connections between neighbouring nodes[25].

Connectome quality control procedure

An initial QC of whole-brain tractography was conducted on a randomly selected subset of STRADL participants (*n* = 50). This involved visual inspection of successful interregional streamlines alongside slices from the FA volume using TrackVis visualisation software (https://trackvis.org). This pilot QC indicated that low streamline counts could serve as a heuristic for identifying major tractography errors or other systematic errors during processing. Based on these findings, QC was performed on a subset of 80 participants, leading to the exclusion of 19 due to aberrant streamlines and sparse cortical connectivity. The final STRADL dataset included 938 connectome participants.

For UKB, we performed a similar manual QC of connectome outputs in a subset of 388 out of 37,436 participants (available imaging sample at time of processing) that were identified as outliers (± 3 SDs) based on the mean edge weights for SC, FA or MD using unthresholded networks. The QC for these outliers involved visual inspection of the successful interregional streamlines alongside slices from the FA volume. Exclusions were made for major tractography failures or problems in the FA volume for which 152 out of 388 (39.2%) outliers were excluded. 50 of these exclusions were due to tracking failures caused by incomplete FreeSurfer segmentations. Other reasons for failure were: signal dropout, warping or distortions in the dMRI (FA) volume; sparse or aberrant connectivity to cortical areas; and tracking failure across the corpus callosum. We observed that a low total number of interregional streamlines (i.e. 3 SDs below the mean SC) was often indicative of tractography error or other systematics errors during processing.

For LBC1936, we performed a manual QC of connectome outputs in a subset of 64 out of 615 participants (wave 2) that were identified as outliers (± 2 SDs) based on the mean edge weights for SC, FA or MD using unthresholded networks. We selected a more stringent outlier criteria than for UKB due to the more elderly sample. As above, the QC for these outliers involved visual inspection of the successful interregional streamlines alongside slices from the FA volume. Exclusions were made for major tractography failures or problems in the FA volume for which 12 out of 614 (18.8%)

outliers were excluded. Four fails were due to problems in dMRI volumes (motion artefacts or poor eddy current correction), while eight fails had aberrant or sparse tractography in cortical areas.

After removing processing failures and quality checking, the STRADL dataset comprised 937 connectome participants (553 female, 384 male, mean age 59.5 years, 26.0–84.0 years), the UKB main sample had 18,642 participants (9,911 female, 8,731 male, mean age 63.7 years, 44.6–81.7 years), and LBC1936 had 603 participants (282 female, 321 male, mean age 72.7 years, 71.0–74.2 years). In addition, a further 18,642 UKB participants (9,946 female, 8,696 male, mean age 64.0, 44.8-82.7 years) were processed as a held-out replication sample.

Cognitive tests

Cognitive test data were collected as described in detail elsewhere: UKB[27], STRADL[2], LBC1936[28]. Although the test batteries varied across cohorts, tests were selected to cover multiple cognitive domains in each, including crystallised and fluid cognitive ability domains. This approach allowed us to estimate a latent factor of *g* that is known to be highly consistent across samples despite having been extracted from different tests[29,30]. The tests used in the present study are detailed below and summarised in Supplementary Tables 2-4.

The ten cognitive tests used for UKB[1,27] were: reaction time (time taken to respond in snap-type computer game; UKB data field 20023), number span (maximum length of number string recalled; field 4282), fluid intelligence (number of 13 verbal and numerical logic questions correct; field 20016), trail making B (time taken to complete an alphanumeric path-making test; field 6350), matrix pattern (number of matrix pattern puzzles solved; field 6373)[31], tower task (number of tower task puzzles solved; field 21003), digit-symbol substitution (number of digit-symbol pairs matched; field 23324)[32], pairs matching (number of incorrect matches in a 6-pair matching game where zero indicates the participant made no mistakes; field 399), prospective memory (test of instruction recall after a delay; field 20018), and paired associates (number of novel word pairs correctly recalled after a delay; field 20197). We coded prospective memory as a binary variable with one indicating success at the first recall attempt. We removed trail making B scores for those who did not complete the test. Reaction time, trail making B, and pairs matching variables were log transformed and then multiplied by -1 so that a higher score indicated better performance.

The five tests used for STRADL[2] were: matrix reasoning (number of puzzles correct;[31], verbal fluency (number of words recalled beginning with C, F and L in 3x1 minute)[33], Mill Hill vocabulary (number of word meanings explained correctly)[34], digit symbol substitution (number of digit-symbol pairs matched)[35], and logical memory (story recall score minus the total number from immediate and delayed tests)[35].

The thirteen tests used for LBC1936[7] were: matrix reasoning (number of puzzles correct)[35], block design (number of puzzles correct in two minutes)[35], spatial span (number of block sequences correct)[36], National Adult Reading Test (number of words pronounced correctly)[37], Wechsler test of adult reading (number of words pronounced correctly)[38], verbal fluency (number of words recalled beginning with C, F and L in 3x1 minute)[39], verbal paired associates (number of novel word pairs matched in recall - total from immediate and delayed tests)[36], logical memory (number of story details recalled out of a total possible of 25 from immediate and delayed tests)[36], digit span backwards (maximum number of a string of numbers recalled in reverse)[35], symbol search (number of symbols correctly detected), digit-symbol substitution (number of digit-symbol pairs matched)[32], inspection time (number of correct responses)[40], and four-choice reaction time(s) (time taken to press the indicated button out of 4 buttons)[41]. Reaction times were multiplied by -1 so that a higher score indicated better performance.

For each cohort, a latent factor of *g* was estimated from these multi-domain cognitive tests using confirmatory factor analysis (CFA) in a structural equation modelling (SEM) framework. For STRADL, a residual covariance was set between Mill Hill vocabulary and digit symbol substitution tests. For LBC, within-domain residual covariances were added for four cognitive domains (visuospatial, crystalised ability, verbal memory, and processing speed) in keeping with previous models [42]. We used a full information maximum likelihood estimator to handle participants with missing test data. Model fits were assessed using the following fit indices: Comparative Fit Index (CFI), Tucker Lewis Index (TLI), Root Mean Square Error of Approximation (RMSEA), and the Root Mean Square Residual (SRMR).

Supplementary results

Estimation of *g* from cognitive tests

For each cohort, we estimated a latent factor of *g* from multi-domain cognitive tests using CFA. Participants without any cognitive test data were excluded from the cognitive analysis, resulting in the exclusion of 1628 participants from UKB. To handle missing test data, we employed a full information maximum likelihood estimator. The number of remaining participants with missing data for at least one cognitive test was: 5206 (30.6%) for UKB, 26 (4.3%) for LBC1936, and 8 (0.9%) for STRADL. All three CFA models resulted in an acceptable fit (all CFI > 0.94; TLI > 0.92; RMSEA < 0.06; SRMR < 0.05; Supplementary Table 1). Consistent with the extensive literature on the positive manifold in *g*[43,44], and previous work using the present data[42], cognitive tests showed a relatively even pattern of loadings across indicators (Supplementary Tables 2-4). For each cohort, all estimated paths to latent *g* were significant ($p < 0.001$).

Associations of *g* with age and education

To evaluate whether *g* estimates showed comparable associations with relevant covariates across cohorts, we examined correlations between *g* scores and variables known to relate to cognitive ability. These variables included age, age at leaving full-time education, duration of education, and highest educational qualification (Supplementary Table 5). Not all variables were available in every cohort. In addition, highest qualification was coded differently in UKB and LBC1936, and duration of education was measured in years in LBC1936 but on an ordinal scale in STRADL (Compulsory = 1; More than compulsory = 2; Post-secondary = 3). Despite the relatively narrow age range in LBC1936 (~3 years), *g* was negatively associated with age in all cohorts (*r* range: −0.36 to −0.10). Furthermore, despite scoring differences, correlations were comparable across cohorts for duration of education ($r = 0.39$ and 0.47) and highest qualification ($r = 0.35$ and 0.55).

Scanner-specific differences in network properties

Overall, network organisation was comparable across cohorts, although expected scanner-related differences were observed in network density and weight distributions (Supplementary Fig. 1). The mean edge weights for LBC1936 networks (all acquired at 1.5T MRI) were found to be lower than in the other two cohorts (both 3T MRI), particularly for FA and MD networks, although these cross-cohort differences were smaller for SC networks. Higher field strength acquisitions are known to identify more white matter connections than lower field strengths and differences in acquisition and pre-processing can also lead to apparent differences in the resulting FA and MD values[45]. Additionally, STRADL data were acquired on two different scanners (Aberdeen: Philips Achieva 3T; Dundee: Siemens 3T Prisma-FIT), but these acquisitions were well-matched, and the diffusion measures were broadly in the same range (mean edge weight range for FA: 0.35–0.48 for Aberdeen, and 0.33–0.46 for Dundee).

Effect of neurological exclusion on network-*g* associations

Our main analyses included all participants. However, we found that applying exclusion for neurological conditions led to some attenuation in *g* associations. In UKB, participants were excluded for any of the following 26 self-reported conditions (UKB codes 20001 and 20002): brain cancer, meningeal cancer, stroke, transient ischaemic attack, subdural haematoma, subarachnoid haemorrhage, neurological trauma, infection of nervous system, brain abscess, encephalitis, meningitis, Guillain-Barré syndrome, other chronic neurological problems, motor neurone disease, multiple sclerosis, Parkinson's disease, epilepsy, head injury, other demyelinating diseases, brain aneurysm, cerebral palsy, brain haemorrhage, spina bifida, ischaemic stroke, meningioma (benign). After excluding 1172 participants (6.3%) with neurological conditions in UKB, global network-*g* associations were modestly attenuated (0.8–7.3%). Information on neurological conditions differed between LBC1936 and UKB. In LBC1936, excluding 76 participants (12.6%) with

neuroradiologically identified stroke weakened the network-*g* associations (attenuation: 2.1–28.2%), with two exceptions: the clustering coefficient with SC increased by 10.5%, and mean edge weight with MD remained null. Across both cohorts, FA and MD measures showed greater attenuation (UKB: 4.8–7.3%; LBC1936: 12.9–28.2%) than SC (UKB: 0.8–1.1%; LBC1936: 2.1–5.5%), though attenuations were broadly similar across network metrics. As neurological exclusion criteria were unavailable for STRADL at the time of analysis and the exclusion criteria differed across the other two cohorts, we conducted all main analyses using the full samples, which also preserved statistical power. Exclusions were applied only for these global associations for comparison.

Influence of brain volume on streamline counts

Our main analyses were based on streamline counts that were uncorrected for grey or white matter volumes. However, there is evidence that uncorrected streamline counts are influenced by head size and more interregional streamlines are identified in large brains than in small brains, given that voxel dimensions are constant across participants. We observed that the mean edge weight for SC networks was strongly correlated with total brain volume ($r = 0.80$ in UKB), but this was not the case for the diffusion tensor-based FA or MD networks ($r < 0.1$). However, it is unclear how best to apply volume correction of streamline counts to avoid overcorrection for volume-driven effects on these streamline weightings[22,46]. Consequently, we investigated node-*g* associations for a variant of streamline count, termed, streamline density (SD), which corrects SC by dividing each edge count by the combined volume of the pair of grey matter ROIs involved. Notably, the node-*g* associations were lower for SD (β range: -0.14 to 0.19; Supplementary Table 8) than for SC (β range: -0.10 to 0.27). However, the meta-analysis of node strength for SD networks found that 28/85 (32.9%) nodes retained significant node-*g* associations compared to 72/85 (84.7%) for SC. The cross-cohort correlations based on the node βs for SD were moderate (r range: 0.34 to 0.48).

Analysis of significant edge-*g* associations

Significant intra-hemisphere connections were considerably more numerous than inter-hemisphere connections with this ratio exceeding 4:1 across all network weightings (Supplementary Table 15). However, across all lobes, the significant intra-lobe connections were fewer than the significant connections to other lobes (Supplementary Table 16). These inter-lobe connections were predominantly ipsilateral rather than contralateral. This suggests that associations of *g* at the lobular level were facilitated more by ipsilateral inter-lobe connectivity than within-lobe connectivity.

The significant network edges obtained from the meta-analyses revealed patterns of strong ($|\beta| > 0.1$) intra- and inter-hemispheric connectivity to all cerebral lobes (SC, FA and MD networks). Strong associations for inter-hemispheric connections were observed, particularly between frontal regions (SC, FA and MD), and between bilateral parietal regions (SC). The inter-hemispheric associations were more widespread in MD networks than SC or FA. For all networks, strong associations were found for the precuneus and insula, which are considered integral to network communication. Strong associations were also found for subcortical connectivity, particularly for the thalamus, caudate, putamen and hippocampus, which mainly involved ipsilateral connectivity for SC networks, but some contralateral cortical connectivity for FA and MD networks. We observed little difference between the β distributions of significant intra- and inter-hemisphere connections (Supplementary Fig. 5A). We avoided conducting statistical tests of differences in these βs, because it would be invalid to test a quantity that has already been selected based on significance. Therefore, the following are treated as descriptive. MD networks appeared to show marginally stronger associations for inter- rather than intra-hemisphere connections. For lobe connectivity, we found little difference between β distributions for significant intra- and inter-lobe edges (Supplementary Fig. 5B). However, for FA and MD networks, the connections involving frontal and parietal nodes tended to have stronger associations compared to those involving subcortical, temporal or occipital nodes (Supplementary Fig. 5B and Supplementary Table 16).

Tables

Supplementary Table 1. Model fit measures for the latent *g* model as estimated by confirmatory factor analysis. A single latent factor of *g* was estimated for each cohort: UKB (*n*=17,014), STRADL (*n*=937), and LBC1936 (*n*=603). The measures are: χ2, degrees of freedom (*df*), Comparative Fit Index (CFI), Tucker Lewis Index (TLI), Root Mean Square Error of Approximation (RMSEA), and the Root Mean Square Residual (SRMR).

Study	X^2	*df*	CFI	TLI	RMSEA	SRMR
UKB	1318.668	35	0.943	0.926	0.046	0.031
STRADL	12.566	4	0.986	0.965	0.048	0.022
LBC1936	155.516	50	0.967	0.949	0.059	0.042

Supplementary Table 2. UKB cognitive tests used in latent *g* model with factor loadings (standardised estimates) for individual tests. All estimated paths to latent *g* were significant ($p < 0.001$).

Test	*n*	Standardised estimate	*SE*
Reaction time	16905	0.352	0.008
Number span	12800	0.462	0.008
Fluid intelligence	16684	0.621	0.007
Trail making B	12084	0.733	0.006
Matrix pattern	12388	0.630	0.007
Tower task	12276	0.564	0.007
Digit-symbol substitution	12394	0.612	0.007
Pairs matching	17014	0.279	0.009
Prospective memory	17007	0.364	0.008
Paired associates	12530	0.451	0.008

Supplementary Table 3. STRADL cognitive tests used in latent *g* model with factor loadings (standardised estimates) for individual tests. All estimated paths to latent *g* were significant ($p < 0.001$).

Test	*n*	Standardised estimate	*SE*
Matrix reasoning	937	0.545	0.031
Verbal fluency	937	0.483	0.031
Mill Hill vocabulary	937	0.721	0.035
Digit symbol substitution	929	0.573	0.039
Logical memory	937	0.447	0.031

Supplementary Table 4. LBC1936 cognitive tests used in latent *g* model with factor loadings (standardised estimates) for individual tests. All estimated paths to latent *g* were significant ($p < 0.001$).

Test	*n*	Standardised estimate	*SE*
Matrix reasoning	602	0.572	0.034
Block design	601	0.595	0.034
Spatial span	602	0.399	0.042
National Adult Reading Test	602	0.666	0.031
Wechsler test of adult reading	602	0.681	0.030
Phonemic verbal fluency	602	0.489	0.038
Verbal paired associates	592	0.543	0.037
Logical memory	602	0.560	0.036
Digit span backwards	603	0.573	0.035
Symbol search	602	0.591	0.035
Digit-symbol substitution	602	0.644	0.031
Inspection time	594	0.378	0.042
Four-choice reaction time (s)	603	0.403	0.041

Supplementary Table 5. Correlations (Pearson's *r*) between *g* scores and demographic variables in each cohort: age, age at leaving full-time education, duration of education, and highest qualification obtained. Note the narrow age range in the LBC1936 cohort (~3 years). Dash indicates that information was not available. All correlations were significant ($p < 0.01$, uncorrected).

Study	Age	Age left full-time education	Duration of education	Highest qualification
UKB	-0.365	0.155	-	0.350
STRADL	-0.102	-	0.391	-
LBC1936	-0.154	0.465	0.465	0.547

Supplementary Table 6. Associations (standardised estimates) between *g* and global network metrics in all cohorts, including the meta-analysis, across weightings (SC, FA, and MD), and across network metrics (mean edge weight, global efficiency, mean clustering coefficient). Standard errors (*SE*) and *p*-values were estimated using Structural Equation Modelling (SEM), and *q*-values were adjusted by False Discovery Rate (FDR).

Study	Weighting	Metric	β	*SE*	*p*	*q*
UKB	SC	Mean edge weight	0.159	0.009	0.000	0.000
UKB	SC	Efficiency	0.152	0.009	0.000	0.000
UKB	SC	Clustering	0.166	0.009	0.000	0.000
UKB	FA	Mean edge weight	0.063	0.008	0.000	0.000
UKB	FA	Efficiency	0.058	0.008	0.000	0.000
UKB	FA	Clustering	0.066	0.008	0.000	0.000
UKB	MD	Mean edge weight	-0.061	0.009	0.000	0.000
UKB	MD	Efficiency	-0.075	0.009	0.000	0.000
UKB	MD	Clustering	-0.061	0.009	0.000	0.000
STRADL	SC	Mean edge weight	0.277	0.036	0.000	0.000
STRADL	SC	Efficiency	0.267	0.037	0.000	0.000
STRADL	SC	Clustering	0.280	0.035	0.000	0.000
STRADL	FA	Mean edge weight	0.293	0.048	0.000	0.000
STRADL	FA	Efficiency	0.300	0.052	0.000	0.000
STRADL	FA	Clustering	0.272	0.049	0.000	0.000
STRADL	MD	Mean edge weight	-0.159	0.060	0.008	0.008
STRADL	MD	Efficiency	-0.216	0.056	0.000	0.000
STRADL	MD	Clustering	-0.214	0.058	0.000	0.000
LBC1936	SC	Mean edge weight	0.179	0.044	0.000	0.000
LBC1936	SC	Efficiency	0.157	0.044	0.000	0.002
LBC1936	SC	Clustering	0.120	0.045	0.007	0.016
LBC1936	FA	Mean edge weight	0.127	0.045	0.005	0.014
LBC1936	FA	Efficiency	0.108	0.045	0.015	0.028
LBC1936	FA	Clustering	0.100	0.045	0.026	0.039
LBC1936	MD	Mean edge weight	-0.006	0.046	0.902	0.902
LBC1936	MD	Efficiency	-0.084	0.046	0.066	0.080
LBC1936	MD	Clustering	-0.082	0.046	0.071	0.080
Meta-analysis	SC	Mean edge weight	0.201	0.037	0.000	0.000
Meta-analysis	SC	Efficiency	0.188	0.037	0.000	0.000
Meta-analysis	SC	Clustering	0.189	0.045	0.000	0.000
Meta-analysis	FA	Mean edge weight	0.155	0.068	0.023	0.027
Meta-analysis	FA	Efficiency	0.149	0.072	0.039	0.039
Meta-analysis	FA	Clustering	0.140	0.062	0.024	0.027
Meta-analysis	MD	Mean edge weight	-0.064	0.026	0.014	0.022
Meta-analysis	MD	Efficiency	-0.112	0.040	0.005	0.011
Meta-analysis	MD	Clustering	-0.106	0.043	0.013	0.022

Supplementary Table 7. Standardised associations between *g* and local efficiency in all cohorts, including the meta-analysis, for 85 FreeSurfer regions in uncorrected SC networks. *SE* and *p*-values were estimated by SEM, and *q*-values were adjusted by FDR.

		UKB				STRADL				LBC1936				Meta-analysis			
**Hemisphere**	**Node**	**β**	***SE***	***p***	***q***	**β**	***SE***	***p***	***q***	**β**	***SE***	***p***	***q***	**β**	***SE***	***p***	***q***
Left	Thalamus	0.172	0.009	0.000	0.000	0.216	0.037	0.000	0.000	0.158	0.044	0.000	0.005	0.174	0.008	0.000	0.000
Left	Caudate	0.056	0.008	0.000	0.000	0.181	0.037	0.000	0.000	0.007	0.045	0.879	0.937	0.081	0.049	0.097	0.106
Left	Putamen	0.139	0.009	0.000	0.000	0.217	0.037	0.000	0.000	0.084	0.045	0.060	0.147	0.148	0.033	0.000	0.000
Left	Pallidum	0.097	0.009	0.000	0.000	0.075	0.037	0.041	0.048	0.101	0.045	0.026	0.084	0.096	0.008	0.000	0.000
Left	Hippocampus	0.091	0.009	0.000	0.000	0.105	0.037	0.005	0.007	0.112	0.045	0.012	0.059	0.092	0.008	0.000	0.000
Left	Amygdala	0.061	0.008	0.000	0.000	0.050	0.037	0.172	0.179	0.072	0.045	0.111	0.219	0.061	0.008	0.000	0.000
Left	Accumbens-area	0.038	0.009	0.000	0.000	0.017	0.037	0.645	0.645	0.021	0.045	0.632	0.768	0.036	0.008	0.000	0.000
Left	Ventral diencephalon	0.079	0.008	0.000	0.000	0.058	0.041	0.155	0.165	0.077	0.045	0.086	0.191	0.078	0.008	0.000	0.000
Left	Banks of superior temporal	0.074	0.008	0.000	0.000	0.143	0.037	0.000	0.000	0.034	0.045	0.450	0.599	0.083	0.024	0.000	0.001
Left	Caudal anterior cingulate	0.063	0.008	0.000	0.000	0.143	0.037	0.000	0.000	0.017	0.045	0.706	0.822	0.075	0.031	0.016	0.020
Left	Caudal middle frontal	0.102	0.009	0.000	0.000	0.187	0.035	0.000	0.000	0.070	0.045	0.118	0.228	0.119	0.031	0.000	0.000
Left	Cuneus	0.043	0.008	0.000	0.000	0.119	0.036	0.001	0.001	0.023	0.045	0.610	0.768	0.059	0.025	0.018	0.022
Left	Entorhinal	0.087	0.008	0.000	0.000	0.137	0.037	0.000	0.000	0.087	0.045	0.052	0.137	0.090	0.009	0.000	0.000
Left	Fusiform	0.101	0.008	0.000	0.000	0.050	0.037	0.170	0.179	0.076	0.045	0.091	0.197	0.091	0.015	0.000	0.000
Left	Inferior parietal	0.142	0.008	0.000	0.000	0.167	0.036	0.000	0.000	0.081	0.045	0.071	0.166	0.141	0.008	0.000	0.000
Left	Inferior temporal	0.117	0.008	0.000	0.000	0.127	0.036	0.000	0.001	0.047	0.045	0.295	0.440	0.115	0.008	0.000	0.000
Left	Isthmus cingulate	0.060	0.009	0.000	0.000	0.177	0.037	0.000	0.000	0.062	0.046	0.174	0.321	0.097	0.038	0.011	0.014
Left	Lateral occipital	0.123	0.008	0.000	0.000	0.147	0.037	0.000	0.000	0.117	0.044	0.008	0.046	0.124	0.008	0.000	0.000
Left	Lateral orbitofrontal	0.082	0.009	0.000	0.000	0.205	0.038	0.000	0.000	-0.051	0.045	0.255	0.428	0.080	0.071	0.260	0.273
Left	Lingual	0.057	0.008	0.000	0.000	0.118	0.039	0.002	0.003	0.058	0.045	0.203	0.360	0.065	0.015	0.000	0.000
Left	Medial orbitofrontal	0.058	0.008	0.000	0.000	0.118	0.037	0.001	0.002	0.014	0.046	0.754	0.855	0.063	0.018	0.000	0.001
Left	Middle temporal	0.113	0.009	0.000	0.000	0.188	0.038	0.000	0.000	0.094	0.045	0.037	0.112	0.127	0.023	0.000	0.000
Left	Parahippocampal	0.096	0.009	0.000	0.000	0.110	0.038	0.004	0.005	0.050	0.045	0.262	0.428	0.095	0.008	0.000	0.000
Left	Paracentral	0.081	0.008	0.000	0.000	0.164	0.035	0.000	0.000	0.109	0.044	0.014	0.064	0.110	0.027	0.000	0.000
Left	Pars opercularis	0.102	0.009	0.000	0.000	0.186	0.038	0.000	0.000	0.044	0.046	0.335	0.483	0.112	0.035	0.002	0.002
Left	Pars orbitalis	0.069	0.009	0.000	0.000	0.093	0.037	0.013	0.016	0.008	0.045	0.857	0.934	0.068	0.008	0.000	0.000
Left	Pars triangularis	0.099	0.009	0.000	0.000	0.159	0.037	0.000	0.000	0.049	0.045	0.280	0.440	0.104	0.021	0.000	0.000
Left	Pericalcarine	0.027	0.008	0.001	0.001	0.079	0.036	0.028	0.033	-0.016	0.045	0.722	0.830	0.028	0.008	0.001	0.001
Left	Postcentral	0.121	0.009	0.000	0.000	0.181	0.036	0.000	0.000	0.125	0.046	0.007	0.040	0.133	0.017	0.000	0.000
Left	Posterior cingulate	0.064	0.008	0.000	0.000	0.120	0.037	0.001	0.002	0.079	0.045	0.075	0.173	0.075	0.016	0.000	0.000
Left	Precentral	0.127	0.009	0.000	0.000	0.274	0.035	0.000	0.000	0.131	0.045	0.003	0.029	0.176	0.048	0.000	0.000
Left	Precuneus	0.112	0.008	0.000	0.000	0.206	0.035	0.000	0.000	0.088	0.045	0.050	0.136	0.135	0.033	0.000	0.000

Left	Rostral anterior cingulate	0.059	0.008	0.000	0.000	0.137	0.037	0.000	0.000	-0.007	0.045	0.882	0.937	0.065	0.036	0.068	0.076
Left	Rostral middle frontal	0.106	0.009	0.000	0.000	0.239	0.037	0.000	0.000	0.003	0.045	0.949	0.974	0.117	0.066	0.074	0.081
Left	Superior frontal	0.142	0.009	0.000	0.000	0.234	0.036	0.000	0.000	0.090	0.045	0.045	0.132	0.156	0.038	0.000	0.000
Left	Superior parietal	0.121	0.009	0.000	0.000	0.188	0.036	0.000	0.000	0.116	0.044	0.009	0.046	0.135	0.020	0.000	0.000
Left	Superior temporal	0.123	0.009	0.000	0.000	0.236	0.037	0.000	0.000	0.022	0.046	0.627	0.768	0.129	0.058	0.027	0.032
Left	Supramarginal	0.122	0.009	0.000	0.000	0.198	0.037	0.000	0.000	0.074	0.045	0.099	0.207	0.132	0.030	0.000	0.000
Left	Frontal pole	0.070	0.008	0.000	0.000	0.158	0.040	0.000	0.000	-0.042	0.045	0.349	0.495	0.064	0.054	0.236	0.251
Left	Temporal pole	0.060	0.008	0.000	0.000	0.089	0.038	0.019	0.023	-0.013	0.046	0.768	0.859	0.059	0.008	0.000	0.000
Left	Transverse temporal	0.081	0.008	0.000	0.000	0.136	0.036	0.000	0.000	0.001	0.045	0.986	0.986	0.078	0.032	0.015	0.019
Left	Insula	0.091	0.008	0.000	0.000	0.148	0.036	0.000	0.000	-0.050	0.045	0.267	0.428	0.067	0.055	0.224	0.241
Right	Thalamus	0.187	0.009	0.000	0.000	0.254	0.037	0.000	0.000	0.168	0.044	0.000	0.003	0.198	0.020	0.000	0.000
Right	Caudate	0.045	0.009	0.000	0.000	0.102	0.039	0.008	0.010	0.018	0.046	0.694	0.819	0.046	0.009	0.000	0.000
Right	Putamen	0.154	0.009	0.000	0.000	0.243	0.036	0.000	0.000	0.160	0.044	0.000	0.005	0.180	0.028	0.000	0.000
Right	Pallidum	0.146	0.009	0.000	0.000	0.100	0.036	0.006	0.008	0.123	0.045	0.006	0.040	0.139	0.013	0.000	0.000
Right	Hippocampus	0.081	0.009	0.000	0.000	0.136	0.036	0.000	0.000	0.173	0.044	0.000	0.002	0.118	0.029	0.000	0.000
Right	Amygdala	0.039	0.009	0.000	0.000	0.041	0.040	0.305	0.312	-0.059	0.045	0.192	0.347	0.018	0.027	0.497	0.503
Right	Accumbens-area	0.064	0.009	0.000	0.000	0.064	0.036	0.079	0.085	0.034	0.045	0.451	0.599	0.063	0.008	0.000	0.000
Right	Ventral diencephalon	0.096	0.008	0.000	0.000	0.103	0.037	0.005	0.007	0.089	0.045	0.047	0.134	0.096	0.008	0.000	0.000
Right	Banks of superior temporal	0.047	0.009	0.000	0.000	0.074	0.037	0.047	0.053	0.100	0.045	0.024	0.084	0.053	0.012	0.000	0.000
Right	Caudal anterior cingulate	0.042	0.008	0.000	0.000	0.145	0.036	0.000	0.000	0.022	0.045	0.620	0.768	0.068	0.035	0.055	0.064
Right	Caudal middle frontal	0.084	0.009	0.000	0.000	0.206	0.034	0.000	0.000	0.140	0.044	0.002	0.015	0.137	0.038	0.000	0.000
Right	Cuneus	0.027	0.008	0.001	0.001	0.145	0.036	0.000	0.000	-0.050	0.045	0.263	0.428	0.042	0.054	0.430	0.440
Right	Entorhinal	0.064	0.008	0.000	0.000	0.064	0.036	0.076	0.083	0.048	0.045	0.294	0.440	0.064	0.008	0.000	0.000
Right	Fusiform	0.082	0.008	0.000	0.000	0.122	0.038	0.001	0.002	0.099	0.045	0.026	0.084	0.085	0.008	0.000	0.000
Right	Inferior parietal	0.130	0.008	0.000	0.000	0.202	0.035	0.000	0.000	0.105	0.044	0.018	0.076	0.144	0.024	0.000	0.000
Right	Inferior temporal	0.107	0.008	0.000	0.000	0.142	0.036	0.000	0.000	0.086	0.045	0.053	0.137	0.108	0.008	0.000	0.000
Right	Isthmus cingulate	0.050	0.009	0.000	0.000	0.107	0.038	0.006	0.007	0.042	0.045	0.359	0.501	0.054	0.011	0.000	0.000
Right	Lateral occipital	0.099	0.008	0.000	0.000	0.182	0.037	0.000	0.000	0.035	0.045	0.436	0.598	0.107	0.037	0.004	0.005
Right	Lateral orbitofrontal	0.086	0.009	0.000	0.000	0.145	0.038	0.000	0.000	-0.002	0.045	0.962	0.974	0.081	0.036	0.027	0.032
Right	Lingual	0.047	0.008	0.000	0.000	0.145	0.037	0.000	0.000	0.046	0.045	0.309	0.452	0.075	0.031	0.017	0.021
Right	Medial orbitofrontal	0.073	0.008	0.000	0.000	0.099	0.036	0.006	0.008	0.011	0.046	0.803	0.886	0.072	0.008	0.000	0.000
Right	Middle temporal	0.115	0.008	0.000	0.000	0.192	0.037	0.000	0.000	0.142	0.045	0.001	0.015	0.139	0.024	0.000	0.000
Right	Parahippocampal	0.085	0.008	0.000	0.000	0.128	0.036	0.000	0.001	0.099	0.045	0.026	0.084	0.088	0.008	0.000	0.000
Right	Paracentral	0.094	0.008	0.000	0.000	0.133	0.036	0.000	0.000	0.121	0.044	0.006	0.040	0.098	0.010	0.000	0.000
Right	Pars opercularis	0.107	0.009	0.000	0.000	0.157	0.037	0.000	0.000	0.071	0.045	0.110	0.219	0.108	0.008	0.000	0.000
Right	Pars orbitalis	0.120	0.009	0.000	0.000	0.131	0.038	0.000	0.001	0.019	0.045	0.674	0.807	0.100	0.029	0.000	0.001
Right	Pars triangularis	0.094	0.009	0.000	0.000	0.148	0.037	0.000	0.000	0.117	0.045	0.009	0.046	0.106	0.016	0.000	0.000

Right	Pericalcarine	0.018	0.008	0.033	0.033	0.067	0.036	0.063	0.070	0.047	0.045	0.294	0.440	0.029	0.016	0.068	0.076
Right	Postcentral	0.129	0.008	0.000	0.000	0.179	0.035	0.000	0.000	0.205	0.044	0.000	0.000	0.157	0.025	0.000	0.000
Right	Posterior cingulate	0.077	0.008	0.000	0.000	0.131	0.038	0.000	0.001	0.030	0.045	0.503	0.658	0.078	0.008	0.000	0.000
Right	Precentral	0.126	0.009	0.000	0.000	0.214	0.036	0.000	0.000	0.201	0.043	0.000	0.000	0.171	0.032	0.000	0.000
Right	Precuneus	0.123	0.008	0.000	0.000	0.186	0.035	0.000	0.000	0.100	0.044	0.025	0.084	0.132	0.018	0.000	0.000
Right	Rostral anterior cingulate	0.052	0.008	0.000	0.000	0.164	0.039	0.000	0.000	-0.053	0.045	0.243	0.422	0.056	0.059	0.346	0.358
Right	Rostral middle frontal	0.120	0.009	0.000	0.000	0.241	0.039	0.000	0.000	0.063	0.045	0.159	0.300	0.142	0.049	0.004	0.005
Right	Superior frontal	0.136	0.009	0.000	0.000	0.256	0.035	0.000	0.000	0.153	0.044	0.000	0.006	0.178	0.038	0.000	0.000
Right	Superior parietal	0.131	0.009	0.000	0.000	0.210	0.035	0.000	0.000	0.127	0.044	0.004	0.032	0.151	0.025	0.000	0.000
Right	Superior temporal	0.129	0.009	0.000	0.000	0.152	0.035	0.000	0.000	0.101	0.046	0.027	0.084	0.129	0.008	0.000	0.000
Right	Supramarginal	0.095	0.009	0.000	0.000	0.108	0.036	0.003	0.004	0.027	0.046	0.554	0.714	0.093	0.008	0.000	0.000
Right	Frontal pole	0.060	0.008	0.000	0.000	0.156	0.038	0.000	0.000	0.002	0.045	0.961	0.974	0.074	0.040	0.068	0.076
Right	Temporal pole	0.046	0.008	0.000	0.000	-0.026	0.036	0.466	0.471	-0.099	0.045	0.026	0.084	-0.018	0.042	0.674	0.674
Right	Transverse temporal	0.068	0.008	0.000	0.000	0.078	0.037	0.032	0.037	-0.006	0.047	0.897	0.941	0.067	0.008	0.000	0.000
Right	Insula	0.088	0.009	0.000	0.000	0.215	0.036	0.000	0.000	0.084	0.045	0.061	0.147	0.127	0.042	0.003	0.004
	Brainstem	0.044	0.009	0.000	0.000	0.069	0.037	0.060	0.067	0.074	0.045	0.100	0.207	0.046	0.008	0.000	0.000

Supplementary Table 8. Standardised associations between *g* and local efficiency in all cohorts, including the meta-analysis, for 85 FreeSurfer regions in volume corrected SD networks. *SE* and *p*-values were estimated by SEM, and *q*-values were adjusted by FDR.

		**UKB**				**STRADL**				**LBC1936**				**Meta-analysis**			
**Hemisphere**	**Node**	**β**	***SE***	***p***	***q***	**β**	***SE***	***p***	***q***	**β**	***SE***	***p***	***q***	**β**	***SE***	***p***	***q***
Left	Thalamus	0.069	0.009	0.000	0.000	0.085	0.037	0.021	0.047	-0.000	0.045	0.998	0.998	0.068	0.008	0.000	0.000
Left	Caudate	-0.008	0.008	0.347	0.404	0.060	0.037	0.101	0.156	-0.105	0.045	0.019	0.317	-0.014	0.043	0.746	0.828
Left	Putamen	0.021	0.008	0.015	0.025	0.089	0.036	0.014	0.037	-0.048	0.045	0.288	0.847	0.024	0.033	0.469	0.594
Left	Pallidum	0.019	0.009	0.027	0.044	0.005	0.037	0.902	0.924	-0.043	0.045	0.344	0.867	0.016	0.009	0.094	0.207
Left	Hippocampus	0.037	0.009	0.000	0.000	0.045	0.038	0.233	0.315	0.037	0.046	0.426	0.867	0.037	0.008	0.000	0.000
Left	Amygdala	-0.002	0.008	0.796	0.836	-0.023	0.036	0.529	0.583	-0.008	0.045	0.858	0.976	-0.003	0.008	0.677	0.777
Left	Accumbens-area	-0.013	0.008	0.113	0.159	-0.048	0.037	0.195	0.268	-0.034	0.045	0.451	0.867	-0.016	0.008	0.052	0.127
Left	Ventral diencephalon	-0.007	0.008	0.415	0.473	-0.032	0.042	0.445	0.504	-0.061	0.045	0.174	0.773	-0.012	0.011	0.293	0.429
Left	Banks of superior temporal	0.013	0.008	0.127	0.169	0.080	0.038	0.037	0.072	-0.035	0.045	0.438	0.867	0.019	0.024	0.414	0.559
Left	Caudal anterior cingulate	0.002	0.008	0.824	0.839	0.081	0.037	0.028	0.062	-0.015	0.045	0.739	0.976	0.019	0.025	0.457	0.594
Left	Caudal middle frontal	0.042	0.008	0.000	0.000	0.134	0.035	0.000	0.001	0.021	0.045	0.644	0.976	0.064	0.032	0.042	0.105
Left	Cuneus	0.002	0.008	0.829	0.839	0.076	0.036	0.037	0.072	-0.038	0.046	0.408	0.867	0.014	0.027	0.612	0.713
Left	Entorhinal	0.047	0.008	0.000	0.000	0.092	0.036	0.011	0.033	0.003	0.045	0.941	0.976	0.048	0.008	0.000	0.000
Left	Fusiform	0.031	0.009	0.000	0.001	-0.036	0.037	0.339	0.412	-0.034	0.046	0.459	0.867	0.000	0.026	0.988	0.988
Left	Inferior parietal	0.081	0.008	0.000	0.000	0.086	0.036	0.018	0.043	-0.004	0.045	0.936	0.976	0.072	0.017	0.000	0.000
Left	Inferior temporal	0.040	0.008	0.000	0.000	0.055	0.037	0.136	0.196	-0.047	0.045	0.299	0.847	0.030	0.020	0.125	0.231
Left	Isthmus cingulate	-0.016	0.009	0.072	0.111	0.097	0.039	0.014	0.037	-0.036	0.045	0.425	0.867	0.012	0.038	0.747	0.828
Left	Lateral occipital	0.063	0.008	0.000	0.000	0.072	0.040	0.068	0.123	0.047	0.045	0.293	0.847	0.063	0.008	0.000	0.000
Left	Lateral orbitofrontal	0.006	0.008	0.480	0.530	0.104	0.039	0.008	0.027	-0.098	0.045	0.030	0.317	0.006	0.054	0.917	0.939
Left	Lingual	0.011	0.008	0.204	0.258	0.071	0.041	0.084	0.147	-0.035	0.046	0.448	0.867	0.012	0.008	0.155	0.268
Left	Medial orbitofrontal	-0.000	0.008	0.983	0.983	0.041	0.037	0.269	0.357	-0.017	0.047	0.719	0.976	0.001	0.008	0.872	0.938
Left	Middle temporal	0.042	0.008	0.000	0.000	0.104	0.042	0.012	0.034	0.004	0.045	0.928	0.976	0.043	0.008	0.000	0.000
Left	Parahippocampal	0.050	0.009	0.000	0.000	0.058	0.038	0.132	0.196	-0.025	0.045	0.572	0.934	0.048	0.008	0.000	0.000
Left	Paracentral	0.011	0.008	0.209	0.258	0.087	0.036	0.017	0.042	0.004	0.045	0.936	0.976	0.028	0.024	0.234	0.349
Left	Pars opercularis	0.039	0.008	0.000	0.000	0.107	0.036	0.003	0.012	-0.031	0.046	0.500	0.904	0.042	0.033	0.202	0.312
Left	Pars orbitalis	0.013	0.008	0.115	0.160	0.020	0.036	0.574	0.618	-0.040	0.045	0.378	0.867	0.012	0.008	0.139	0.246
Left	Pars triangularis	0.034	0.008	0.000	0.000	0.091	0.036	0.011	0.033	-0.010	0.045	0.830	0.976	0.039	0.017	0.019	0.057
Left	Pericalcarine	-0.011	0.008	0.209	0.258	0.038	0.037	0.303	0.391	-0.070	0.045	0.120	0.773	-0.010	0.008	0.216	0.327
Left	Postcentral	0.047	0.008	0.000	0.000	0.105	0.036	0.004	0.013	0.012	0.045	0.792	0.976	0.052	0.015	0.001	0.003
Left	Posterior cingulate	-0.010	0.008	0.248	0.301	0.052	0.037	0.154	0.219	0.012	0.045	0.784	0.976	0.006	0.019	0.750	0.828
Left	Precentral	0.047	0.008	0.000	0.000	0.186	0.035	0.000	0.000	0.017	0.045	0.699	0.976	0.083	0.050	0.098	0.207
Left	Precuneus	0.032	0.008	0.000	0.000	0.119	0.038	0.002	0.010	-0.041	0.046	0.375	0.867	0.038	0.041	0.347	0.491

Left	Rostral anterior cingulate	0.004	0.008	0.588	0.633	0.062	0.037	0.094	0.150	-0.016	0.046	0.724	0.976	0.009	0.013	0.470	0.594
Left	Rostral middle frontal	0.042	0.008	0.000	0.000	0.150	0.037	0.000	0.001	-0.044	0.046	0.332	0.867	0.051	0.053	0.329	0.475
Left	Superior frontal	0.063	0.009	0.000	0.000	0.137	0.036	0.000	0.001	-0.022	0.046	0.629	0.976	0.063	0.041	0.123	0.231
Left	Superior parietal	0.048	0.008	0.000	0.000	0.108	0.035	0.002	0.011	0.026	0.045	0.568	0.934	0.056	0.017	0.001	0.005
Left	Superior temporal	0.034	0.008	0.000	0.000	0.159	0.040	0.000	0.001	-0.068	0.045	0.134	0.773	0.043	0.063	0.494	0.599
Left	Supramarginal	0.050	0.008	0.000	0.000	0.131	0.037	0.000	0.003	-0.005	0.045	0.920	0.976	0.060	0.034	0.074	0.176
Left	Frontal pole	0.034	0.008	0.000	0.000	0.095	0.041	0.021	0.047	-0.060	0.046	0.195	0.773	0.027	0.038	0.489	0.599
Left	Temporal pole	0.013	0.008	0.124	0.169	0.023	0.037	0.535	0.583	-0.064	0.045	0.153	0.773	0.011	0.008	0.180	0.289
Left	Transverse temporal	0.030	0.008	0.000	0.001	0.068	0.037	0.063	0.117	-0.044	0.045	0.331	0.867	0.030	0.008	0.000	0.001
Left	Insula	0.017	0.008	0.036	0.058	0.076	0.037	0.037	0.072	-0.117	0.045	0.009	0.226	-0.004	0.053	0.943	0.954
Right	Thalamus	0.108	0.008	0.000	0.000	0.162	0.037	0.000	0.000	0.054	0.045	0.227	0.803	0.110	0.014	0.000	0.000
Right	Caudate	0.015	0.009	0.084	0.126	0.039	0.039	0.314	0.393	-0.052	0.047	0.267	0.841	0.014	0.008	0.102	0.207
Right	Putamen	0.040	0.009	0.000	0.000	0.106	0.037	0.004	0.015	0.016	0.046	0.727	0.976	0.050	0.019	0.010	0.033
Right	Pallidum	0.084	0.008	0.000	0.000	0.036	0.036	0.314	0.393	-0.027	0.045	0.551	0.934	0.043	0.032	0.180	0.289
Right	Hippocampus	0.030	0.009	0.000	0.001	0.065	0.038	0.084	0.147	0.073	0.045	0.105	0.773	0.036	0.011	0.002	0.007
Right	Amygdala	-0.008	0.008	0.320	0.378	-0.036	0.038	0.345	0.413	-0.128	0.044	0.004	0.160	-0.048	0.034	0.164	0.279
Right	Accumbens-area	0.017	0.008	0.040	0.063	0.019	0.037	0.612	0.651	-0.001	0.046	0.980	0.992	0.017	0.008	0.038	0.097
Right	Ventral diencephalon	0.007	0.009	0.417	0.473	-0.004	0.038	0.917	0.928	-0.065	0.045	0.146	0.773	-0.002	0.015	0.916	0.939
Right	Banks of superior temporal	-0.020	0.009	0.021	0.035	-0.031	0.039	0.434	0.498	0.019	0.045	0.671	0.976	-0.019	0.008	0.021	0.060
Right	Caudal anterior cingulate	0.003	0.008	0.715	0.760	0.080	0.038	0.035	0.072	-0.007	0.046	0.886	0.976	0.020	0.024	0.407	0.559
Right	Caudal middle frontal	0.014	0.008	0.101	0.145	0.137	0.035	0.000	0.001	0.059	0.045	0.191	0.773	0.064	0.038	0.093	0.207
Right	Cuneus	-0.006	0.008	0.492	0.537	0.120	0.037	0.001	0.008	-0.098	0.045	0.028	0.317	0.007	0.060	0.914	0.939
Right	Entorhinal	0.034	0.008	0.000	0.000	0.001	0.036	0.984	0.984	-0.020	0.045	0.655	0.976	0.024	0.015	0.112	0.216
Right	Fusiform	0.019	0.009	0.023	0.038	0.036	0.038	0.339	0.412	0.013	0.045	0.776	0.976	0.020	0.008	0.014	0.044
Right	Inferior parietal	0.059	0.008	0.000	0.000	0.110	0.036	0.002	0.011	-0.007	0.045	0.869	0.976	0.059	0.023	0.009	0.031
Right	Inferior temporal	0.033	0.008	0.000	0.000	0.066	0.040	0.095	0.150	-0.018	0.046	0.694	0.976	0.033	0.008	0.000	0.000
Right	Isthmus cingulate	-0.015	0.009	0.088	0.130	0.044	0.041	0.287	0.375	-0.051	0.045	0.254	0.830	-0.014	0.008	0.102	0.207
Right	Lateral occipital	0.032	0.008	0.000	0.000	0.133	0.042	0.002	0.009	-0.058	0.045	0.200	0.773	0.036	0.051	0.475	0.594
Right	Lateral orbitofrontal	0.011	0.008	0.196	0.253	0.050	0.039	0.194	0.268	-0.062	0.046	0.181	0.773	0.010	0.008	0.201	0.312
Right	Lingual	0.006	0.008	0.478	0.530	0.097	0.039	0.014	0.037	-0.039	0.046	0.389	0.867	0.021	0.035	0.549	0.657
Right	Medial orbitofrontal	0.021	0.008	0.013	0.024	0.030	0.037	0.421	0.497	0.027	0.046	0.560	0.934	0.022	0.008	0.008	0.030
Right	Middle temporal	0.040	0.008	0.000	0.000	0.113	0.041	0.006	0.020	0.038	0.045	0.399	0.867	0.052	0.019	0.007	0.027
Right	Parahippocampal	0.039	0.008	0.000	0.000	0.055	0.037	0.134	0.196	0.004	0.045	0.921	0.976	0.039	0.008	0.000	0.000
Right	Paracentral	0.034	0.008	0.000	0.000	0.078	0.036	0.032	0.068	0.061	0.046	0.182	0.773	0.041	0.012	0.001	0.004
Right	Pars opercularis	0.054	0.008	0.000	0.000	0.074	0.037	0.044	0.084	0.004	0.045	0.936	0.976	0.053	0.008	0.000	0.000
Right	Pars orbitalis	0.061	0.008	0.000	0.000	0.061	0.037	0.093	0.150	-0.028	0.045	0.540	0.934	0.047	0.021	0.026	0.070
Right	Pars triangularis	0.031	0.008	0.000	0.001	0.062	0.037	0.090	0.150	0.053	0.045	0.240	0.816	0.033	0.008	0.000	0.000

Right	Pericalcarine	-0.014	0.008	0.084	0.126	0.029	0.037	0.431	0.498	-0.005	0.045	0.917	0.976	-0.012	0.008	0.133	0.241
Right	Postcentral	0.070	0.008	0.000	0.000	0.107	0.036	0.003	0.012	0.113	0.044	0.011	0.226	0.078	0.014	0.000	0.000
Right	Posterior cingulate	0.021	0.008	0.011	0.020	0.061	0.038	0.109	0.166	-0.036	0.045	0.424	0.867	0.021	0.008	0.010	0.034
Right	Precentral	0.049	0.008	0.000	0.000	0.131	0.036	0.000	0.002	0.087	0.045	0.051	0.478	0.080	0.027	0.003	0.013
Right	Precuneus	0.056	0.008	0.000	0.000	0.117	0.038	0.002	0.010	-0.006	0.046	0.902	0.976	0.059	0.026	0.023	0.064
Right	Rostral anterior cingulate	0.012	0.008	0.164	0.214	0.149	0.040	0.000	0.002	-0.065	0.046	0.154	0.773	0.032	0.059	0.591	0.698
Right	Rostral middle frontal	0.056	0.008	0.000	0.000	0.146	0.041	0.000	0.002	-0.011	0.047	0.821	0.976	0.065	0.040	0.101	0.207
Right	Superior frontal	0.057	0.009	0.000	0.000	0.158	0.036	0.000	0.000	0.061	0.045	0.177	0.773	0.088	0.033	0.007	0.027
Right	Superior parietal	0.062	0.008	0.000	0.000	0.133	0.035	0.000	0.001	0.012	0.045	0.795	0.976	0.071	0.029	0.014	0.043
Right	Superior temporal	0.059	0.008	0.000	0.000	0.062	0.037	0.095	0.150	-0.019	0.045	0.669	0.976	0.053	0.013	0.000	0.000
Right	Supramarginal	0.028	0.008	0.001	0.001	0.017	0.037	0.652	0.676	-0.101	0.045	0.023	0.317	-0.010	0.038	0.788	0.859
Right	Frontal pole	0.026	0.008	0.002	0.004	0.091	0.039	0.019	0.044	-0.017	0.046	0.717	0.976	0.032	0.020	0.106	0.209
Right	Temporal pole	0.013	0.008	0.125	0.169	-0.091	0.036	0.011	0.033	-0.137	0.044	0.002	0.157	-0.064	0.046	0.167	0.279
Right	Transverse temporal	0.029	0.008	0.001	0.001	0.025	0.036	0.499	0.559	-0.057	0.045	0.210	0.776	0.016	0.019	0.408	0.559
Right	Insula	0.009	0.008	0.277	0.332	0.108	0.037	0.003	0.012	-0.032	0.045	0.474	0.877	0.029	0.038	0.450	0.594
	Brainstem	-0.002	0.008	0.818	0.839	0.016	0.036	0.651	0.676	0.002	0.045	0.967	0.990	-0.001	0.008	0.910	0.939

Supplementary Table 9. Standardised associations between *g* and local efficiency in all cohorts, including the meta-analysis, for 85 FreeSurfer regions in FA networks. *SE* and *p*-values were estimated by SEM, and *q*-values were adjusted by FDR.

		UKB				STRADL				LBC1936				Meta-analysis			
**Hemisphere**	**Node**	**β**	***SE***	***p***	***q***	**β**	***SE***	***p***	***q***	**β**	***SE***	***p***	***q***	**β**	***SE***	***p***	***q***
Left	Thalamus	0.039	0.008	0.000	0.000	0.269	0.055	0.000	0.000	0.083	0.045	0.066	0.137	0.123	0.068	0.072	0.078
Left	Caudate	0.048	0.009	0.000	0.000	0.237	0.050	0.000	0.000	0.097	0.045	0.030	0.090	0.119	0.055	0.031	0.045
Left	Putamen	0.057	0.008	0.000	0.000	0.297	0.054	0.000	0.000	0.099	0.045	0.027	0.090	0.144	0.072	0.046	0.055
Left	Pallidum	0.044	0.008	0.000	0.000	0.206	0.054	0.000	0.000	0.067	0.045	0.137	0.207	0.095	0.047	0.042	0.053
Left	Hippocampus	0.057	0.008	0.000	0.000	0.187	0.044	0.000	0.000	0.074	0.045	0.104	0.181	0.099	0.039	0.012	0.029
Left	Amygdala	0.037	0.008	0.000	0.000	0.124	0.044	0.005	0.006	0.080	0.046	0.078	0.151	0.066	0.027	0.015	0.031
Left	Accumbens-area	0.039	0.008	0.000	0.000	0.109	0.043	0.011	0.013	0.001	0.045	0.987	0.987	0.043	0.014	0.003	0.010
Left	Ventral diencephalon	0.061	0.008	0.000	0.000	0.249	0.059	0.000	0.000	0.070	0.045	0.120	0.200	0.116	0.056	0.039	0.051
Left	Banks of superior temporal	0.061	0.008	0.000	0.000	0.216	0.049	0.000	0.000	0.083	0.045	0.066	0.137	0.112	0.046	0.015	0.031
Left	Caudal anterior cingulate	0.043	0.008	0.000	0.000	0.090	0.041	0.027	0.030	-0.007	0.045	0.885	0.906	0.043	0.008	0.000	0.000
Left	Caudal middle frontal	0.059	0.009	0.000	0.000	0.219	0.046	0.000	0.000	0.068	0.045	0.128	0.204	0.110	0.050	0.028	0.043
Left	Cuneus	0.058	0.008	0.000	0.000	0.157	0.038	0.000	0.000	0.042	0.046	0.366	0.414	0.082	0.033	0.013	0.029
Left	Entorhinal	0.048	0.008	0.000	0.000	0.120	0.039	0.002	0.002	0.113	0.044	0.011	0.062	0.081	0.027	0.003	0.010
Left	Fusiform	0.079	0.008	0.000	0.000	0.212	0.046	0.000	0.000	0.086	0.046	0.059	0.128	0.119	0.041	0.003	0.011
Left	Inferior parietal	0.065	0.009	0.000	0.000	0.221	0.050	0.000	0.000	0.118	0.045	0.009	0.056	0.125	0.045	0.006	0.017
Left	Inferior temporal	0.044	0.008	0.000	0.000	0.216	0.050	0.000	0.000	0.074	0.045	0.103	0.181	0.103	0.051	0.043	0.053
Left	Isthmus cingulate	0.038	0.008	0.000	0.000	0.247	0.050	0.000	0.000	0.087	0.045	0.053	0.128	0.117	0.062	0.059	0.066
Left	Lateral occipital	0.071	0.009	0.000	0.000	0.223	0.050	0.000	0.000	0.137	0.045	0.003	0.056	0.133	0.045	0.003	0.010
Left	Lateral orbitofrontal	0.067	0.009	0.000	0.000	0.132	0.041	0.001	0.002	0.074	0.045	0.097	0.175	0.076	0.016	0.000	0.000
Left	Lingual	0.070	0.008	0.000	0.000	0.183	0.042	0.000	0.000	0.063	0.046	0.172	0.244	0.100	0.036	0.005	0.016
Left	Medial orbitofrontal	0.061	0.009	0.000	0.000	0.095	0.045	0.035	0.038	0.041	0.045	0.361	0.414	0.062	0.008	0.000	0.000
Left	Middle temporal	0.046	0.008	0.000	0.000	0.210	0.051	0.000	0.000	0.092	0.045	0.041	0.115	0.106	0.048	0.026	0.041
Left	Parahippocampal	0.071	0.008	0.000	0.000	0.210	0.040	0.000	0.000	0.054	0.045	0.232	0.295	0.109	0.047	0.021	0.036
Left	Paracentral	0.031	0.008	0.000	0.000	0.200	0.047	0.000	0.000	0.127	0.044	0.004	0.056	0.111	0.051	0.030	0.044
Left	Pars opercularis	0.056	0.009	0.000	0.000	0.198	0.048	0.000	0.000	0.081	0.045	0.071	0.144	0.103	0.042	0.014	0.031
Left	Pars orbitalis	0.065	0.009	0.000	0.000	0.187	0.042	0.000	0.000	0.076	0.045	0.089	0.164	0.103	0.037	0.006	0.017
Left	Pars triangularis	0.064	0.009	0.000	0.000	0.226	0.045	0.000	0.000	0.057	0.045	0.200	0.266	0.111	0.053	0.034	0.047
Left	Pericalcarine	0.068	0.008	0.000	0.000	0.175	0.041	0.000	0.000	0.087	0.045	0.056	0.128	0.102	0.033	0.002	0.008
Left	Postcentral	0.048	0.008	0.000	0.000	0.234	0.049	0.000	0.000	0.108	0.045	0.015	0.076	0.122	0.055	0.025	0.040
Left	Posterior cingulate	0.038	0.008	0.000	0.000	0.171	0.045	0.000	0.000	0.086	0.045	0.054	0.128	0.089	0.039	0.024	0.040
Left	Precentral	0.049	0.008	0.000	0.000	0.227	0.050	0.000	0.000	0.121	0.045	0.007	0.056	0.124	0.052	0.018	0.035
Left	Precuneus	0.043	0.008	0.000	0.000	0.223	0.051	0.000	0.000	0.104	0.045	0.020	0.081	0.115	0.053	0.029	0.044

Left	Rostral anterior cingulate	0.049	0.008	0.000	0.000	0.080	0.039	0.039	0.042	-0.050	0.045	0.263	0.319	0.033	0.032	0.303	0.311
Left	Rostral middle frontal	0.052	0.009	0.000	0.000	0.200	0.044	0.000	0.000	0.105	0.044	0.018	0.081	0.111	0.044	0.012	0.029
Left	Superior frontal	0.063	0.009	0.000	0.000	0.228	0.047	0.000	0.000	0.097	0.045	0.029	0.090	0.122	0.049	0.013	0.029
Left	Superior parietal	0.051	0.008	0.000	0.000	0.234	0.051	0.000	0.000	0.118	0.045	0.009	0.056	0.126	0.053	0.019	0.035
Left	Superior temporal	0.049	0.008	0.000	0.000	0.243	0.052	0.000	0.000	0.056	0.045	0.216	0.278	0.110	0.061	0.071	0.077
Left	Supramarginal	0.058	0.008	0.000	0.000	0.222	0.050	0.000	0.000	0.051	0.045	0.257	0.317	0.104	0.052	0.047	0.055
Left	Frontal pole	0.043	0.009	0.000	0.000	0.157	0.041	0.000	0.000	0.059	0.045	0.190	0.260	0.079	0.035	0.022	0.037
Left	Temporal pole	0.030	0.008	0.000	0.000	0.068	0.039	0.079	0.084	0.069	0.045	0.127	0.204	0.036	0.012	0.002	0.010
Left	Transverse temporal	0.038	0.008	0.000	0.000	0.131	0.045	0.004	0.004	-0.013	0.045	0.778	0.827	0.049	0.035	0.153	0.159
Left	Insula	0.055	0.008	0.000	0.000	0.178	0.045	0.000	0.000	0.035	0.045	0.445	0.491	0.084	0.040	0.037	0.050
Right	Thalamus	0.049	0.008	0.000	0.000	0.261	0.054	0.000	0.000	0.047	0.045	0.298	0.357	0.113	0.068	0.096	0.101
Right	Caudate	0.049	0.008	0.000	0.000	0.135	0.045	0.002	0.003	0.068	0.045	0.130	0.204	0.070	0.024	0.004	0.013
Right	Putamen	0.053	0.008	0.000	0.000	0.238	0.051	0.000	0.000	0.086	0.045	0.056	0.128	0.118	0.055	0.032	0.045
Right	Pallidum	0.057	0.008	0.000	0.000	0.143	0.050	0.004	0.005	0.024	0.045	0.598	0.652	0.059	0.008	0.000	0.000
Right	Hippocampus	0.051	0.008	0.000	0.000	0.132	0.042	0.002	0.002	0.080	0.045	0.075	0.149	0.075	0.025	0.003	0.010
Right	Amygdala	0.032	0.009	0.000	0.000	0.019	0.039	0.625	0.625	-0.011	0.045	0.802	0.841	0.030	0.008	0.000	0.003
Right	Accumbens-area	0.025	0.008	0.002	0.003	0.047	0.038	0.225	0.230	0.016	0.045	0.721	0.776	0.026	0.008	0.001	0.007
Right	Ventral diencephalon	0.043	0.008	0.000	0.000	0.190	0.058	0.001	0.001	0.046	0.045	0.305	0.360	0.080	0.042	0.056	0.063
Right	Banks of superior temporal	0.040	0.008	0.000	0.000	0.202	0.049	0.000	0.000	0.062	0.045	0.168	0.242	0.094	0.049	0.054	0.063
Right	Caudal anterior cingulate	0.044	0.008	0.000	0.000	0.139	0.042	0.001	0.001	-0.004	0.045	0.934	0.945	0.058	0.036	0.105	0.110
Right	Caudal middle frontal	0.078	0.009	0.000	0.000	0.186	0.041	0.000	0.000	0.099	0.045	0.028	0.090	0.113	0.033	0.001	0.004
Right	Cuneus	0.055	0.008	0.000	0.000	0.122	0.039	0.002	0.002	0.104	0.045	0.021	0.081	0.080	0.024	0.001	0.005
Right	Entorhinal	0.062	0.009	0.000	0.000	0.082	0.037	0.028	0.031	0.079	0.045	0.082	0.154	0.063	0.008	0.000	0.000
Right	Fusiform	0.073	0.008	0.000	0.000	0.156	0.041	0.000	0.000	0.102	0.045	0.024	0.090	0.098	0.025	0.000	0.001
Right	Inferior parietal	0.061	0.008	0.000	0.000	0.213	0.049	0.000	0.000	0.090	0.045	0.045	0.115	0.113	0.045	0.011	0.029
Right	Inferior temporal	0.048	0.008	0.000	0.000	0.213	0.048	0.000	0.000	0.067	0.045	0.136	0.207	0.102	0.050	0.041	0.052
Right	Isthmus cingulate	0.043	0.008	0.000	0.000	0.218	0.049	0.000	0.000	0.098	0.045	0.028	0.090	0.111	0.051	0.030	0.044
Right	Lateral occipital	0.071	0.008	0.000	0.000	0.182	0.048	0.000	0.000	0.120	0.045	0.007	0.056	0.111	0.033	0.001	0.005
Right	Lateral orbitofrontal	0.059	0.008	0.000	0.000	0.140	0.040	0.000	0.001	0.052	0.045	0.248	0.310	0.076	0.024	0.001	0.007
Right	Lingual	0.061	0.008	0.000	0.000	0.228	0.043	0.000	0.000	0.058	0.045	0.196	0.265	0.112	0.055	0.039	0.051
Right	Medial orbitofrontal	0.050	0.009	0.000	0.000	0.064	0.040	0.106	0.111	-0.009	0.045	0.836	0.867	0.049	0.009	0.000	0.000
Right	Middle temporal	0.040	0.008	0.000	0.000	0.231	0.049	0.000	0.000	0.064	0.045	0.155	0.231	0.106	0.058	0.070	0.077
Right	Parahippocampal	0.068	0.008	0.000	0.000	0.173	0.041	0.000	0.000	0.125	0.045	0.005	0.056	0.112	0.034	0.001	0.005
Right	Paracentral	0.044	0.008	0.000	0.000	0.252	0.047	0.000	0.000	0.150	0.044	0.001	0.056	0.142	0.061	0.021	0.036
Right	Pars opercularis	0.073	0.009	0.000	0.000	0.176	0.043	0.000	0.000	0.091	0.045	0.043	0.115	0.103	0.030	0.001	0.005
Right	Pars orbitalis	0.079	0.009	0.000	0.000	0.196	0.041	0.000	0.000	0.120	0.044	0.007	0.056	0.124	0.036	0.001	0.004
Right	Pars triangularis	0.075	0.009	0.000	0.000	0.204	0.044	0.000	0.000	0.091	0.045	0.042	0.115	0.116	0.039	0.003	0.010

Right	Pericalcarine	0.073	0.008	0.000	0.000	0.145	0.043	0.001	0.001	0.120	0.045	0.007	0.056	0.098	0.024	0.000	0.001
Right	Postcentral	0.058	0.008	0.000	0.000	0.242	0.047	0.000	0.000	0.112	0.045	0.013	0.068	0.131	0.055	0.017	0.033
Right	Posterior cingulate	0.045	0.008	0.000	0.000	0.171	0.044	0.000	0.000	0.045	0.045	0.318	0.371	0.081	0.039	0.039	0.051
Right	Precentral	0.048	0.008	0.000	0.000	0.206	0.048	0.000	0.000	0.121	0.045	0.006	0.056	0.116	0.047	0.014	0.029
Right	Precuneus	0.049	0.008	0.000	0.000	0.230	0.050	0.000	0.000	0.118	0.045	0.008	0.056	0.124	0.053	0.019	0.035
Right	Rostral anterior cingulate	0.045	0.008	0.000	0.000	0.098	0.038	0.009	0.011	-0.124	0.044	0.005	0.056	0.010	0.064	0.882	0.882
Right	Rostral middle frontal	0.059	0.009	0.000	0.000	0.191	0.042	0.000	0.000	0.103	0.044	0.020	0.081	0.110	0.040	0.006	0.016
Right	Superior frontal	0.063	0.009	0.000	0.000	0.215	0.046	0.000	0.000	0.107	0.045	0.017	0.080	0.120	0.045	0.008	0.020
Right	Superior parietal	0.059	0.008	0.000	0.000	0.263	0.052	0.000	0.000	0.118	0.045	0.009	0.056	0.138	0.060	0.020	0.036
Right	Superior temporal	0.040	0.008	0.000	0.000	0.197	0.048	0.000	0.000	0.070	0.045	0.120	0.200	0.094	0.046	0.043	0.053
Right	Supramarginal	0.052	0.008	0.000	0.000	0.228	0.048	0.000	0.000	0.056	0.045	0.210	0.275	0.107	0.055	0.054	0.063
Right	Frontal pole	0.041	0.009	0.000	0.000	0.105	0.040	0.009	0.011	0.061	0.045	0.176	0.246	0.054	0.018	0.003	0.010
Right	Temporal pole	0.020	0.008	0.016	0.016	0.020	0.038	0.606	0.613	0.063	0.045	0.162	0.237	0.022	0.008	0.007	0.019
Right	Transverse temporal	0.001	0.008	0.890	0.890	0.052	0.037	0.160	0.166	-0.034	0.045	0.444	0.491	0.002	0.008	0.765	0.774
Right	Insula	0.034	0.008	0.000	0.000	0.182	0.048	0.000	0.000	0.090	0.045	0.045	0.115	0.093	0.044	0.034	0.047
	Brainstem	0.037	0.008	0.000	0.000	0.149	0.047	0.001	0.002	0.085	0.045	0.058	0.128	0.078	0.034	0.020	0.036

Supplementary Table 10. Standardised associations between *g* and local efficiency in all cohorts, including the meta-analysis, for 85 FreeSurfer regions in MD networks. *SE* and *p*-values were estimated by SEM, and *q*-values were adjusted by FDR.

		UKB				STRADL				LBC1936				Meta-analysis			
**Hemisphere**	**Node**	**β**	***SE***	***p***	***q***	**β**	***SE***	***p***	***q***	**β**	***SE***	***p***	***q***	**β**	***SE***	***p***	***q***
Left	Thalamus	-0.076	0.010	0.000	0.000	-0.238	0.055	0.000	0.000	-0.141	0.045	0.002	0.076	-0.140	0.047	0.003	0.011
Left	Caudate	-0.085	0.010	0.000	0.000	-0.199	0.050	0.000	0.000	-0.116	0.045	0.010	0.078	-0.120	0.032	0.000	0.001
Left	Putamen	-0.068	0.009	0.000	0.000	-0.229	0.055	0.000	0.000	-0.102	0.045	0.025	0.091	-0.121	0.046	0.009	0.019
Left	Pallidum	-0.061	0.009	0.000	0.000	-0.224	0.058	0.000	0.000	-0.060	0.046	0.194	0.235	-0.104	0.049	0.032	0.043
Left	Hippocampus	-0.040	0.009	0.000	0.000	-0.119	0.046	0.009	0.011	-0.046	0.046	0.319	0.348	-0.053	0.020	0.009	0.019
Left	Amygdala	-0.035	0.009	0.000	0.000	-0.122	0.046	0.008	0.011	-0.068	0.045	0.130	0.181	-0.060	0.025	0.018	0.029
Left	Accumbens-area	-0.021	0.008	0.013	0.014	-0.080	0.040	0.048	0.052	0.002	0.045	0.971	0.971	-0.023	0.008	0.006	0.017
Left	Ventral diencephalon	-0.045	0.009	0.000	0.000	-0.174	0.053	0.001	0.002	-0.104	0.046	0.022	0.091	-0.094	0.038	0.013	0.023
Left	Banks of superior temporal	-0.048	0.009	0.000	0.000	-0.199	0.055	0.000	0.001	-0.066	0.046	0.149	0.197	-0.092	0.043	0.032	0.043
Left	Caudal anterior cingulate	-0.057	0.009	0.000	0.000	-0.156	0.052	0.003	0.004	0.022	0.045	0.631	0.647	-0.061	0.044	0.173	0.177
Left	Caudal middle frontal	-0.076	0.009	0.000	0.000	-0.211	0.050	0.000	0.000	-0.077	0.045	0.088	0.145	-0.113	0.041	0.006	0.017
Left	Cuneus	-0.014	0.009	0.117	0.121	-0.147	0.060	0.014	0.017	-0.107	0.045	0.017	0.091	-0.074	0.042	0.081	0.089
Left	Entorhinal	-0.036	0.008	0.000	0.000	-0.042	0.044	0.332	0.345	-0.094	0.045	0.036	0.098	-0.038	0.008	0.000	0.000
Left	Fusiform	-0.047	0.009	0.000	0.000	-0.194	0.054	0.000	0.001	-0.074	0.046	0.103	0.154	-0.093	0.042	0.026	0.036
Left	Inferior parietal	-0.052	0.009	0.000	0.000	-0.198	0.058	0.001	0.001	-0.116	0.045	0.010	0.078	-0.107	0.042	0.010	0.021
Left	Inferior temporal	-0.048	0.009	0.000	0.000	-0.195	0.055	0.000	0.001	-0.100	0.046	0.028	0.091	-0.101	0.042	0.016	0.026
Left	Isthmus cingulate	-0.048	0.009	0.000	0.000	-0.233	0.059	0.000	0.000	-0.119	0.046	0.009	0.078	-0.121	0.053	0.022	0.032
Left	Lateral occipital	-0.031	0.009	0.001	0.001	-0.191	0.059	0.001	0.002	-0.125	0.045	0.006	0.076	-0.102	0.048	0.034	0.043
Left	Lateral orbitofrontal	-0.050	0.009	0.000	0.000	-0.144	0.046	0.002	0.002	-0.100	0.045	0.026	0.091	-0.084	0.029	0.004	0.014
Left	Lingual	-0.018	0.009	0.049	0.052	-0.192	0.057	0.001	0.001	-0.077	0.046	0.093	0.147	-0.084	0.050	0.093	0.098
Left	Medial orbitofrontal	-0.059	0.009	0.000	0.000	-0.110	0.043	0.011	0.014	-0.056	0.045	0.212	0.247	-0.061	0.009	0.000	0.000
Left	Middle temporal	-0.056	0.009	0.000	0.000	-0.212	0.056	0.000	0.000	-0.101	0.045	0.027	0.091	-0.110	0.044	0.013	0.023
Left	Parahippocampal	-0.006	0.009	0.478	0.483	-0.187	0.050	0.000	0.000	-0.042	0.046	0.364	0.392	-0.071	0.054	0.189	0.191
Left	Paracentral	-0.099	0.009	0.000	0.000	-0.273	0.055	0.000	0.000	-0.115	0.045	0.011	0.080	-0.152	0.052	0.003	0.012
Left	Pars opercularis	-0.071	0.009	0.000	0.000	-0.223	0.052	0.000	0.000	-0.087	0.045	0.054	0.111	-0.118	0.045	0.009	0.019
Left	Pars orbitalis	-0.069	0.009	0.000	0.000	-0.172	0.047	0.000	0.001	-0.085	0.045	0.059	0.117	-0.097	0.029	0.001	0.005
Left	Pars triangularis	-0.078	0.009	0.000	0.000	-0.226	0.051	0.000	0.000	-0.067	0.045	0.136	0.187	-0.116	0.047	0.013	0.023
Left	Pericalcarine	-0.019	0.009	0.034	0.036	-0.147	0.055	0.008	0.010	-0.060	0.045	0.184	0.230	-0.060	0.036	0.093	0.098
Left	Postcentral	-0.080	0.009	0.000	0.000	-0.228	0.054	0.000	0.000	-0.092	0.045	0.044	0.106	-0.122	0.043	0.004	0.015
Left	Posterior cingulate	-0.090	0.009	0.000	0.000	-0.259	0.056	0.000	0.000	-0.096	0.046	0.035	0.098	-0.139	0.051	0.006	0.018
Left	Precentral	-0.081	0.009	0.000	0.000	-0.249	0.053	0.000	0.000	-0.085	0.045	0.062	0.117	-0.130	0.051	0.011	0.022
Left	Precuneus	-0.058	0.009	0.000	0.000	-0.248	0.059	0.000	0.000	-0.112	0.045	0.014	0.090	-0.128	0.054	0.018	0.029

Left	Rostral anterior cingulate	-0.055	0.009	0.000	0.000	-0.140	0.044	0.001	0.002	-0.059	0.045	0.184	0.230	-0.073	0.023	0.002	0.007
Left	Rostral middle frontal	-0.072	0.009	0.000	0.000	-0.181	0.048	0.000	0.000	-0.102	0.045	0.022	0.091	-0.106	0.032	0.001	0.005
Left	Superior frontal	-0.094	0.009	0.000	0.000	-0.223	0.051	0.000	0.000	-0.097	0.045	0.030	0.091	-0.128	0.038	0.001	0.004
Left	Superior parietal	-0.060	0.009	0.000	0.000	-0.230	0.057	0.000	0.000	-0.123	0.045	0.007	0.076	-0.125	0.048	0.010	0.021
Left	Superior temporal	-0.048	0.009	0.000	0.000	-0.232	0.057	0.000	0.000	-0.090	0.046	0.049	0.107	-0.112	0.053	0.034	0.043
Left	Supramarginal	-0.059	0.009	0.000	0.000	-0.221	0.054	0.000	0.000	-0.071	0.046	0.119	0.169	-0.107	0.048	0.026	0.036
Left	Frontal pole	-0.044	0.009	0.000	0.000	-0.134	0.046	0.003	0.005	-0.086	0.046	0.060	0.117	-0.074	0.028	0.007	0.019
Left	Temporal pole	-0.042	0.009	0.000	0.000	-0.083	0.047	0.076	0.080	-0.082	0.045	0.068	0.126	-0.046	0.011	0.000	0.000
Left	Transverse temporal	-0.031	0.009	0.000	0.001	-0.266	0.060	0.000	0.000	-0.019	0.046	0.687	0.695	-0.098	0.076	0.200	0.200
Left	Insula	-0.061	0.009	0.000	0.000	-0.199	0.055	0.000	0.001	-0.075	0.045	0.097	0.150	-0.099	0.039	0.011	0.021
Right	Thalamus	-0.068	0.009	0.000	0.000	-0.259	0.059	0.000	0.000	-0.090	0.046	0.050	0.107	-0.129	0.056	0.022	0.032
Right	Caudate	-0.084	0.009	0.000	0.000	-0.235	0.048	0.000	0.000	-0.088	0.045	0.049	0.107	-0.129	0.047	0.006	0.017
Right	Putamen	-0.069	0.009	0.000	0.000	-0.228	0.056	0.000	0.000	-0.081	0.046	0.074	0.130	-0.116	0.047	0.013	0.023
Right	Pallidum	-0.069	0.009	0.000	0.000	-0.205	0.058	0.000	0.001	-0.031	0.046	0.506	0.525	-0.092	0.045	0.039	0.048
Right	Hippocampus	-0.028	0.009	0.001	0.001	-0.122	0.050	0.015	0.018	-0.049	0.046	0.290	0.325	-0.049	0.025	0.048	0.056
Right	Amygdala	-0.038	0.009	0.000	0.000	-0.035	0.039	0.374	0.383	-0.076	0.045	0.091	0.145	-0.039	0.008	0.000	0.000
Right	Accumbens-area	-0.039	0.009	0.000	0.000	-0.076	0.039	0.048	0.052	-0.064	0.045	0.153	0.197	-0.042	0.008	0.000	0.000
Right	Ventral diencephalon	-0.052	0.009	0.000	0.000	-0.231	0.059	0.000	0.000	-0.101	0.046	0.027	0.091	-0.115	0.051	0.023	0.032
Right	Banks of superior temporal	-0.043	0.009	0.000	0.000	-0.234	0.062	0.000	0.000	-0.059	0.046	0.202	0.238	-0.100	0.056	0.073	0.082
Right	Caudal anterior cingulate	-0.073	0.009	0.000	0.000	-0.164	0.052	0.002	0.002	-0.035	0.045	0.446	0.469	-0.079	0.020	0.000	0.001
Right	Caudal middle frontal	-0.082	0.009	0.000	0.000	-0.179	0.046	0.000	0.000	-0.056	0.045	0.220	0.252	-0.099	0.030	0.001	0.005
Right	Cuneus	-0.028	0.009	0.001	0.001	-0.075	0.054	0.165	0.173	-0.120	0.044	0.007	0.076	-0.061	0.030	0.044	0.052
Right	Entorhinal	-0.032	0.008	0.000	0.000	-0.016	0.039	0.687	0.696	-0.099	0.045	0.026	0.091	-0.034	0.008	0.000	0.000
Right	Fusiform	-0.029	0.009	0.001	0.001	-0.119	0.052	0.023	0.027	-0.095	0.046	0.039	0.103	-0.064	0.030	0.035	0.043
Right	Inferior parietal	-0.058	0.009	0.000	0.000	-0.204	0.059	0.001	0.001	-0.109	0.046	0.017	0.091	-0.108	0.041	0.008	0.019
Right	Inferior temporal	-0.038	0.009	0.000	0.000	-0.160	0.056	0.004	0.006	-0.092	0.046	0.045	0.106	-0.080	0.035	0.024	0.033
Right	Isthmus cingulate	-0.046	0.009	0.000	0.000	-0.203	0.062	0.001	0.002	-0.100	0.046	0.030	0.091	-0.100	0.043	0.021	0.032
Right	Lateral occipital	-0.026	0.009	0.003	0.004	-0.138	0.061	0.025	0.028	-0.124	0.045	0.006	0.076	-0.080	0.040	0.044	0.052
Right	Lateral orbitofrontal	-0.058	0.009	0.000	0.000	-0.170	0.044	0.000	0.000	-0.079	0.045	0.075	0.130	-0.093	0.033	0.005	0.017
Right	Lingual	-0.017	0.009	0.057	0.060	-0.122	0.060	0.042	0.047	-0.091	0.046	0.047	0.107	-0.059	0.035	0.088	0.096
Right	Medial orbitofrontal	-0.051	0.009	0.000	0.000	-0.110	0.040	0.007	0.009	-0.058	0.045	0.197	0.236	-0.057	0.013	0.000	0.000
Right	Middle temporal	-0.041	0.009	0.000	0.000	-0.193	0.058	0.001	0.001	-0.078	0.046	0.091	0.145	-0.090	0.042	0.034	0.043
Right	Parahippocampal	-0.004	0.008	0.657	0.657	-0.153	0.059	0.009	0.012	-0.077	0.047	0.101	0.154	-0.063	0.044	0.150	0.155
Right	Paracentral	-0.101	0.009	0.000	0.000	-0.257	0.054	0.000	0.000	-0.110	0.045	0.015	0.091	-0.146	0.046	0.002	0.006
Right	Pars opercularis	-0.082	0.009	0.000	0.000	-0.219	0.048	0.000	0.000	-0.071	0.045	0.119	0.169	-0.117	0.044	0.007	0.019
Right	Pars orbitalis	-0.066	0.009	0.000	0.000	-0.183	0.046	0.000	0.000	-0.058	0.045	0.194	0.235	-0.095	0.036	0.009	0.019
Right	Pars triangularis	-0.078	0.009	0.000	0.000	-0.195	0.050	0.000	0.000	-0.034	0.045	0.447	0.469	-0.097	0.041	0.019	0.029

Right	Pericalcarine	-0.013	0.009	0.120	0.123	-0.121	0.058	0.037	0.042	-0.129	0.045	0.004	0.076	-0.075	0.042	0.077	0.086
Right	Postcentral	-0.080	0.009	0.000	0.000	-0.243	0.059	0.000	0.000	-0.082	0.046	0.074	0.130	-0.124	0.048	0.010	0.021
Right	Posterior cingulate	-0.075	0.009	0.000	0.000	-0.191	0.054	0.000	0.001	-0.080	0.046	0.079	0.134	-0.101	0.031	0.001	0.005
Right	Precentral	-0.086	0.009	0.000	0.000	-0.246	0.055	0.000	0.000	-0.068	0.046	0.139	0.188	-0.125	0.051	0.015	0.024
Right	Precuneus	-0.063	0.009	0.000	0.000	-0.257	0.060	0.000	0.000	-0.129	0.045	0.004	0.076	-0.137	0.055	0.012	0.023
Right	Rostral anterior cingulate	-0.052	0.009	0.000	0.000	-0.109	0.042	0.009	0.011	-0.084	0.044	0.058	0.117	-0.065	0.017	0.000	0.001
Right	Rostral middle frontal	-0.082	0.009	0.000	0.000	-0.204	0.045	0.000	0.000	-0.100	0.045	0.024	0.091	-0.120	0.036	0.001	0.005
Right	Superior frontal	-0.097	0.009	0.000	0.000	-0.221	0.048	0.000	0.000	-0.091	0.045	0.044	0.106	-0.128	0.038	0.001	0.004
Right	Superior parietal	-0.065	0.009	0.000	0.000	-0.249	0.060	0.000	0.000	-0.134	0.045	0.003	0.076	-0.137	0.052	0.009	0.019
Right	Superior temporal	-0.039	0.009	0.000	0.000	-0.214	0.059	0.000	0.001	-0.104	0.046	0.023	0.091	-0.106	0.050	0.033	0.043
Right	Supramarginal	-0.057	0.009	0.000	0.000	-0.230	0.059	0.000	0.000	-0.052	0.046	0.257	0.292	-0.103	0.053	0.050	0.058
Right	Frontal pole	-0.041	0.009	0.000	0.000	-0.137	0.042	0.001	0.002	-0.092	0.045	0.042	0.106	-0.078	0.031	0.011	0.021
Right	Temporal pole	-0.037	0.009	0.000	0.000	-0.002	0.039	0.955	0.955	-0.096	0.045	0.031	0.091	-0.038	0.008	0.000	0.000
Right	Transverse temporal	-0.020	0.009	0.019	0.021	-0.137	0.045	0.002	0.003	-0.047	0.045	0.297	0.328	-0.058	0.035	0.092	0.098
Right	Insula	-0.055	0.009	0.000	0.000	-0.177	0.056	0.001	0.002	-0.073	0.046	0.110	0.161	-0.085	0.033	0.009	0.019
	Brainstem	-0.042	0.008	0.000	0.000	-0.091	0.046	0.049	0.053	-0.065	0.045	0.151	0.197	-0.044	0.008	0.000	0.000

Supplementary Table 11. Summary of standardised β coefficients for associations between *g* and nodal local efficiency across 85 network nodes, shown for each cohort and the meta-analysis for four network weightings (SC, SD, FA, and MD). Estimates were obtained using SEM, and n_{node} and n_{nodeFDR} indicate the number of significant nodes before and after FDR correction ($p < 0.05$).

Study	Weighting	Mean	Median	Min	Max	n_{node}	$n_{\textit{node}\text{FDR}}$
UKB	SC	0.090	0.088	0.018	0.187	85	85
UKB	SD	0.028	0.030	-0.020	0.108	54	52
UKB	FA	0.052	0.051	0.001	0.079	84	84
UKB	MD	-0.054	-0.055	-0.101	-0.004	80	79
STRADL	SC	0.146	0.145	-0.026	0.274	75	74
STRADL	SD	0.071	0.076	-0.091	0.186	45	38
STRADL	FA	0.179	0.196	0.019	0.297	79	79
STRADL	MD	-0.179	-0.194	-0.273	-0.002	79	76
LBC1936	SC	0.062	0.070	-0.099	0.205	31	17
LBC1936	SD	-0.019	-0.018	-0.137	0.113	8	0
LBC1936	FA	0.072	0.080	-0.124	0.150	33	0
LBC1936	MD	-0.083	-0.085	-0.141	0.022	39	0
Meta-analysis	SC	0.098	0.095	-0.018	0.198	72	72
Meta-analysis	SD	0.029	0.030	-0.064	0.110	34	28
Meta-analysis	FA	0.094	0.103	0.002	0.144	72	62
Meta-analysis	MD	-0.093	-0.098	-0.152	-0.023	73	70

Supplementary Table 12. Correlations (Pearson's r) between paired node-*g* β coefficients across cerebral hemispheres in all cohorts, including the meta-analysis, and across network weightings ($p < 0.001$).

Study	Weighting	*r*	*p*
UKB	SC	0.880	0.000
UKB	SD	0.683	0.000
UKB	FA	0.694	0.000
UKB	MD	0.937	0.000
STRADL	SC	0.698	0.000
STRADL	SD	0.563	0.000
STRADL	FA	0.822	0.000
STRADL	MD	0.819	0.000
LBC1936	SC	0.662	0.000
LBC1936	SD	0.414	0.006
LBC1936	FA	0.761	0.000
LBC1936	MD	0.653	0.000
Meta-analysis	SC	0.839	0.000
Meta-analysis	SD	0.663	0.000
Meta-analysis	FA	0.865	0.000
Meta-analysis	MD	0.879	0.000

Supplementary Table 13. List of grey matter regions (Desikan-Killiany atlas) and abbreviations.

Region	Abbreviation	Lobe/Grouping
Caudal middle frontal	CaMF	Frontal
Frontal pole	FPo	Frontal
Lateral orbitofrontal	LOF	Frontal
Medial orbitofrontal	MedOr	Frontal
Paracentral	PaC	Frontal
Pars opercularis	ParOp	Frontal
Pars orbitalis	ParOr	Frontal
Pars triangularis	ParTr	Frontal
Precentral	PrC	Frontal
Rostral middle frontal	RosMF	Frontal
Superior frontal	SupF	Frontal
Caudal anterior cingulate	CaACg	Cingulate
Isthmus cingulate	IsCg	Cingulate
Posterior cingulate	PosCg	Cingulate
Rostral anterior cingulate	RosACg	Cingulate
Insula	Ins	-
Banks of superior temporal	bSTS	Temporal
Entorhinal	Ent	Temporal
Fusiform	Fus	Temporal
Inferior temporal	InfT	Temporal
Middle temporal	MT	Temporal
Parahippocampal	PaHip	Temporal
Superior temporal	SupT	Temporal
Temporal pole	TPo	Temporal
Transverse temporal	TrT	Temporal
Inferior parietal	InfP	Parietal
Postcentral	PosC	Parietal
Precuneus	PrCun	Parietal
Superior parietal	SupP	Parietal
Supramarginal	SuMar	Parietal
Cuneus	Cun	Occipital
Lateral occipital	LOc	Occipital
Lingual	Lin	Occipital
Pericalcarine	PerCa	Occipital
Nucleus accumbens	NAcc	Subcortical
Amygdala	Amg	Subcortical
Caudate nucleus	CaN	Subcortical
Hippocampus	Hip	Subcortical
Pallidum	Pal	Subcortical
Putamen	Put	Subcortical
Thalamus	Tha	Subcortical
Ventral diencephalon	VDC	Subcortical
Brainstem	BSt	-

Supplementary Table 14. Summary of standardised β coefficients for associations between *g* and 818 network edges in all cohorts, including the meta-analysis, for three weightings (SC, FA, and MD). Estimates were obtained using SEM, and n_{edge} and n_{edgeFDR} indicate the number of significant edges before and after FDR correction ($p < 0.05$).

Study	Weighting	Mean	Median	Min	Max	n_{edge}	n_{edgeFDR}
UKB	SC	0.029	0.028	-0.050	0.100	572	554
UKB	FA	0.032	0.032	-0.033	0.091	643	637
UKB	MD	-0.038	-0.036	-0.104	0.024	619	609
STRADL	SC	0.043	0.044	-0.091	0.191	243	109
STRADL	FA	0.086	0.087	-0.068	0.240	471	409
STRADL	MD	-0.078	-0.076	-0.278	0.124	389	320
LBC1936	SC	0.018	0.018	-0.152	0.229	109	16
LBC1936	FA	0.039	0.039	-0.146	0.194	148	4
LBC1936	MD	-0.021	-0.023	-0.219	0.162	119	7
Meta-analysis	SC	0.030	0.029	-0.071	0.114	434	391
Meta-analysis	FA	0.046	0.046	-0.034	0.134	503	433
Meta-analysis	MD	-0.043	-0.039	-0.146	0.053	383	325

Supplementary Table 15. Summary of β coefficients by inter- and intra-hemisphere connectivity for significant edge-*g* associations ($p < 0.05$, FDR), derived from the separate meta-analyses of SC, FA, and MD networks. Modules include: whole-brain (all connections), inter-hemisphere (bilateral), intra-hemisphere (ipsilateral), and intra-hemisphere (left or right only) connections.

Study	Weighting	Connectivity	n_{edge}	Mean	Median	Min	Max
UKB	SC	Whole-brain	554	0.040	0.039	-0.050	0.100
UKB	SC	Inter-hemisphere	104	0.037	0.039	-0.050	0.087
UKB	SC	Intra-hemisphere	447	0.041	0.040	-0.049	0.100
UKB	SC	Intra-hemisphere (left)	226	0.040	0.037	-0.049	0.100
UKB	SC	Intra-hemisphere (right)	221	0.043	0.042	-0.049	0.097
UKB	FA	Whole-brain	637	0.040	0.038	-0.033	0.091
UKB	FA	Inter-hemisphere	101	0.030	0.028	-0.018	0.063
UKB	FA	Intra-hemisphere	532	0.042	0.042	-0.033	0.091
UKB	FA	Intra-hemisphere (left)	271	0.040	0.040	-0.027	0.084
UKB	FA	Intra-hemisphere (right)	261	0.043	0.043	-0.033	0.091
UKB	MD	Whole-brain	609	-0.049	-0.047	-0.104	0.024
UKB	MD	Inter-hemisphere	125	-0.048	-0.043	-0.104	0.018
UKB	MD	Intra-hemisphere	480	-0.050	-0.048	-0.104	0.024
UKB	MD	Intra-hemisphere (left)	251	-0.048	-0.047	-0.095	0.023
UKB	MD	Intra-hemisphere (right)	229	-0.052	-0.049	-0.104	0.024
STRADL	SC	Whole-brain	109	0.119	0.114	0.097	0.191
STRADL	SC	Inter-hemisphere	29	0.117	0.110	0.097	0.191
STRADL	SC	Intra-hemisphere	79	0.120	0.116	0.098	0.179
STRADL	SC	Intra-hemisphere (left)	37	0.120	0.116	0.098	0.168
STRADL	SC	Intra-hemisphere (right)	42	0.120	0.116	0.099	0.179
STRADL	FA	Whole-brain	409	0.128	0.121	0.082	0.240
STRADL	FA	Inter-hemisphere	106	0.127	0.123	0.085	0.217
STRADL	FA	Intra-hemisphere	301	0.128	0.121	0.082	0.240
STRADL	FA	Intra-hemisphere (left)	161	0.133	0.125	0.082	0.240
STRADL	FA	Intra-hemisphere (right)	140	0.122	0.116	0.083	0.208
STRADL	MD	Whole-brain	320	-0.153	-0.156	-0.278	0.124
STRADL	MD	Inter-hemisphere	47	-0.137	-0.162	-0.223	0.124
STRADL	MD	Intra-hemisphere	273	-0.156	-0.156	-0.278	0.120
STRADL	MD	Intra-hemisphere (left)	145	-0.158	-0.154	-0.243	-0.095
STRADL	MD	Intra-hemisphere (right)	128	-0.155	-0.156	-0.278	0.120
LBC1936	SC	Whole-brain	16	0.090	0.157	-0.152	0.229
LBC1936	SC	Inter-hemisphere	1	0.152	0.152	0.152	0.152
LBC1936	SC	Intra-hemisphere	14	0.081	0.157	-0.152	0.229
LBC1936	SC	Intra-hemisphere (left)	6	0.054	0.151	-0.152	0.168
LBC1936	SC	Intra-hemisphere (right)	8	0.101	0.166	-0.152	0.229
LBC1936	FA	Whole-brain	4	0.182	0.180	0.172	0.194
LBC1936	FA	Inter-hemisphere	1	0.172	0.172	0.172	0.172
LBC1936	FA	Intra-hemisphere	3	0.185	0.184	0.176	0.194
LBC1936	FA	Intra-hemisphere (left)	1	0.194	0.194	0.194	0.194
LBC1936	FA	Intra-hemisphere (right)	2	0.180	0.180	0.176	0.184
LBC1936	MD	Whole-brain	7	-0.127	-0.164	-0.219	0.162
LBC1936	MD	Inter-hemisphere	4	-0.180	-0.172	-0.219	-0.159
LBC1936	MD	Intra-hemisphere	3	-0.056	-0.162	-0.166	0.162
LBC1936	MD	Intra-hemisphere (left)	1	-0.162	-0.162	-0.162	-0.162
LBC1936	MD	Intra-hemisphere (right)	2	-0.002	-0.002	-0.166	0.162
Meta-analysis	SC	Whole-brain	391	0.046	0.044	-0.071	0.109
Meta-analysis	SC	Inter-hemisphere	75	0.044	0.045	-0.049	0.104
Meta-analysis	SC	Intra-hemisphere	315	0.046	0.044	-0.071	0.109
Meta-analysis	SC	Intra-hemisphere (left)	158	0.044	0.040	-0.050	0.103
Meta-analysis	SC	Intra-hemisphere (right)	157	0.049	0.049	-0.071	0.109
Meta-analysis	FA	Whole-brain	433	0.053	0.051	-0.033	0.134
Meta-analysis	FA	Inter-hemisphere	54	0.052	0.050	0.019	0.111

Meta-analysis	FA	Intra-hemisphere	378	0.053	0.051	-0.033	0.134
Meta-analysis	FA	Intra-hemisphere (left)	180	0.053	0.051	-0.026	0.134
Meta-analysis	FA	Intra-hemisphere (right)	198	0.053	0.053	-0.033	0.117
Meta-analysis	MD	Whole-brain	325	-0.062	-0.060	-0.146	0.021
Meta-analysis	MD	Inter-hemisphere	50	-0.063	-0.050	-0.146	-0.020
Meta-analysis	MD	Intra-hemisphere	272	-0.063	-0.061	-0.139	0.021
Meta-analysis	MD	Intra-hemisphere (left)	141	-0.060	-0.059	-0.121	0.021
Meta-analysis	MD	Intra-hemisphere (right)	131	-0.065	-0.063	-0.139	-0.019

Supplementary Table 16. Summary of β coefficients by intra- and inter-lobe connectivity for significant edge-*g* associations ($p < 0.05$, FDR), derived from the separate meta-analyses of SC, FA, and MD networks. Inter-lobe connectivity includes regions outside the designated lobe.

Study	Weighting	Lobe	Connectivity	n_{edge}	Mean	Median	Min	Max
UKB	SC	Frontal	Intra	57	0.038	0.037	0.020	0.067
UKB	SC	Frontal	Inter	160	0.041	0.037	-0.034	0.097
UKB	SC	Cingulate	Intra	7	0.036	0.029	0.027	0.068
UKB	SC	Cingulate	Inter	40	0.019	0.026	-0.050	0.059
UKB	SC	Insula	Intra	0	-	-	-	-
UKB	SC	Insula	Inter	34	0.031	0.034	-0.035	0.086
UKB	SC	Temporal	Intra	27	0.042	0.042	-0.022	0.087
UKB	SC	Temporal	Inter	155	0.042	0.042	-0.031	0.089
UKB	SC	Parietal	Intra	28	0.048	0.043	0.018	0.087
UKB	SC	Parietal	Inter	178	0.044	0.043	-0.032	0.093
UKB	SC	Occipital	Intra	0	-	-	-	-
UKB	SC	Occipital	Inter	44	0.041	0.043	-0.024	0.100
UKB	SC	Subcortical	Intra	33	0.030	0.032	-0.049	0.081
UKB	SC	Subcortical	Inter	193	0.043	0.042	-0.050	0.100
UKB	FA	Frontal	Intra	72	0.042	0.041	0.018	0.069
UKB	FA	Frontal	Inter	185	0.043	0.042	0.017	0.091
UKB	FA	Cingulate	Intra	5	0.025	0.024	0.018	0.040
UKB	FA	Cingulate	Inter	53	0.032	0.030	0.018	0.048
UKB	FA	Insula	Intra	0	-	-	-	-
UKB	FA	Insula	Inter	42	0.029	0.031	-0.033	0.064
UKB	FA	Temporal	Intra	29	0.033	0.033	-0.020	0.059
UKB	FA	Temporal	Inter	178	0.037	0.036	-0.018	0.074
UKB	FA	Parietal	Intra	29	0.044	0.043	0.017	0.081
UKB	FA	Parietal	Inter	201	0.042	0.041	-0.018	0.091
UKB	FA	Occipital	Intra	12	0.046	0.043	0.028	0.062
UKB	FA	Occipital	Inter	71	0.049	0.048	0.017	0.084
UKB	FA	Subcortical	Intra	25	0.027	0.028	-0.029	0.081
UKB	FA	Subcortical	Inter	200	0.039	0.037	-0.033	0.084
UKB	MD	Frontal	Intra	73	-0.059	-0.062	-0.096	-0.018
UKB	MD	Frontal	Inter	202	-0.064	-0.064	-0.104	-0.017
UKB	MD	Cingulate	Intra	11	-0.036	-0.035	-0.063	-0.022
UKB	MD	Cingulate	Inter	59	-0.046	-0.043	-0.085	-0.018
UKB	MD	Insula	Intra	0	-	-	-	-
UKB	MD	Insula	Inter	48	-0.041	-0.038	-0.087	0.018
UKB	MD	Temporal	Intra	20	-0.025	-0.027	-0.037	0.018
UKB	MD	Temporal	Inter	144	-0.036	-0.035	-0.073	0.024
UKB	MD	Parietal	Intra	30	-0.047	-0.049	-0.078	-0.019
UKB	MD	Parietal	Inter	200	-0.050	-0.050	-0.098	-0.018
UKB	MD	Occipital	Intra	1	-0.018	-0.018	-0.018	-0.018
UKB	MD	Occipital	Inter	32	-0.022	-0.022	-0.037	0.024
UKB	MD	Subcortical	Intra	27	-0.036	-0.033	-0.093	0.023
UKB	MD	Subcortical	Inter	209	-0.053	-0.050	-0.104	0.018
STRADL	SC	Frontal	Intra	17	0.115	0.108	0.098	0.162
STRADL	SC	Frontal	Inter	35	0.122	0.119	0.099	0.166
STRADL	SC	Cingulate	Intra	3	0.156	0.155	0.122	0.191
STRADL	SC	Cingulate	Inter	10	0.126	0.116	0.100	0.179
STRADL	SC	Insula	Intra	0	-	-	-	-
STRADL	SC	Insula	Inter	10	0.113	0.112	0.099	0.128
STRADL	SC	Temporal	Intra	2	0.108	0.108	0.106	0.111
STRADL	SC	Temporal	Inter	20	0.114	0.106	0.097	0.157
STRADL	SC	Parietal	Intra	7	0.110	0.107	0.100	0.125
STRADL	SC	Parietal	Inter	45	0.121	0.116	0.097	0.168
STRADL	SC	Occipital	Intra	1	0.100	0.100	0.100	0.100

STRADL	SC	Occipital	Inter	5	0.115	0.112	0.106	0.123
STRADL	SC	Subcortical	Intra	3	0.141	0.127	0.116	0.178
STRADL	SC	Subcortical	Inter	27	0.117	0.110	0.098	0.179
STRADL	FA	Frontal	Intra	43	0.126	0.119	0.086	0.240
STRADL	FA	Frontal	Inter	118	0.129	0.125	0.082	0.203
STRADL	FA	Cingulate	Intra	1	0.094	0.094	0.094	0.094
STRADL	FA	Cingulate	Inter	37	0.123	0.119	0.082	0.203
STRADL	FA	Insula	Intra	0	-	-	-	-
STRADL	FA	Insula	Inter	18	0.114	0.117	0.088	0.158
STRADL	FA	Temporal	Intra	9	0.102	0.096	0.084	0.160
STRADL	FA	Temporal	Inter	129	0.129	0.124	0.083	0.190
STRADL	FA	Parietal	Intra	21	0.119	0.112	0.085	0.196
STRADL	FA	Parietal	Inter	154	0.130	0.124	0.083	0.225
STRADL	FA	Occipital	Intra	2	0.114	0.114	0.087	0.141
STRADL	FA	Occipital	Inter	43	0.134	0.114	0.084	0.236
STRADL	FA	Subcortical	Intra	9	0.119	0.112	0.100	0.154
STRADL	FA	Subcortical	Inter	149	0.133	0.124	0.083	0.236
STRADL	MD	Frontal	Intra	49	-0.155	-0.161	-0.236	-0.094
STRADL	MD	Frontal	Inter	97	-0.159	-0.162	-0.229	0.091
STRADL	MD	Cingulate	Intra	3	-0.178	-0.172	-0.202	-0.160
STRADL	MD	Cingulate	Inter	37	-0.159	-0.157	-0.216	-0.101
STRADL	MD	Insula	Intra	0	-	-	-	-
STRADL	MD	Insula	Inter	21	-0.150	-0.147	-0.238	-0.102
STRADL	MD	Temporal	Intra	7	-0.148	-0.155	-0.194	-0.106
STRADL	MD	Temporal	Inter	64	-0.143	-0.147	-0.243	0.120
STRADL	MD	Parietal	Intra	17	-0.124	-0.149	-0.220	0.124
STRADL	MD	Parietal	Inter	104	-0.161	-0.168	-0.278	0.110
STRADL	MD	Occipital	Intra	1	-0.110	-0.110	-0.110	-0.110
STRADL	MD	Occipital	Inter	17	-0.116	-0.143	-0.229	0.120
STRADL	MD	Subcortical	Intra	11	-0.128	-0.131	-0.189	-0.097
STRADL	MD	Subcortical	Inter	124	-0.163	-0.168	-0.278	0.110
LBC1936	SC	Frontal	Intra	1	0.165	0.165	0.165	0.165
LBC1936	SC	Frontal	Inter	3	0.062	0.168	-0.150	0.168
LBC1936	SC	Cingulate	Intra	1	0.158	0.158	0.158	0.158
LBC1936	SC	Cingulate	Inter	2	-0.151	-0.151	-0.152	-0.150
LBC1936	SC	Insula	Intra	0	-	-	-	-
LBC1936	SC	Insula	Inter	1	-0.150	-0.150	-0.150	-0.150
LBC1936	SC	Temporal	Intra	0	-	-	-	-
LBC1936	SC	Temporal	Inter	1	0.156	0.156	0.156	0.156
LBC1936	SC	Parietal	Intra	2	0.160	0.160	0.146	0.174
LBC1936	SC	Parietal	Inter	2	0.160	0.160	0.152	0.168
LBC1936	SC	Occipital	Intra	0	-	-	-	-
LBC1936	SC	Occipital	Inter	1	0.152	0.152	0.152	0.152
LBC1936	SC	Subcortical	Intra	5	0.121	0.158	-0.152	0.229
LBC1936	SC	Subcortical	Inter	4	0.005	0.003	-0.152	0.168
LBC1936	FA	Frontal	Intra	1	0.172	0.172	0.172	0.172
LBC1936	FA	Frontal	Inter	2	0.189	0.189	0.184	0.194
LBC1936	FA	Cingulate	Intra	0	-	-	-	-
LBC1936	FA	Cingulate	Inter	0	-	-	-	-
LBC1936	FA	Insula	Intra	0	-	-	-	-
LBC1936	FA	Insula	Inter	0	-	-	-	-
LBC1936	FA	Temporal	Intra	1	0.176	0.176	0.176	0.176
LBC1936	FA	Temporal	Inter	0	-	-	-	-
LBC1936	FA	Parietal	Intra	0	-	-	-	-
LBC1936	FA	Parietal	Inter	0	-	-	-	-
LBC1936	FA	Occipital	Intra	0	-	-	-	-
LBC1936	FA	Occipital	Inter	0	-	-	-	-
LBC1936	FA	Subcortical	Intra	0	-	-	-	-

LBC1936	FA	Subcortical	Inter	2	0.189	0.189	0.184	0.194
LBC1936	MD	Frontal	Intra	1	-0.164	-0.164	-0.164	-0.164
LBC1936	MD	Frontal	Inter	3	-0.079	-0.180	-0.219	0.162
LBC1936	MD	Cingulate	Intra	0	-	-	-	-
LBC1936	MD	Cingulate	Inter	1	-0.219	-0.219	-0.219	-0.219
LBC1936	MD	Insula	Intra	0	-	-	-	-
LBC1936	MD	Insula	Inter	0	-	-	-	-
LBC1936	MD	Temporal	Intra	0	-	-	-	-
LBC1936	MD	Temporal	Inter	0	-	-	-	-
LBC1936	MD	Parietal	Intra	1	-0.166	-0.166	-0.166	-0.166
LBC1936	MD	Parietal	Inter	1	-0.162	-0.162	-0.162	-0.162
LBC1936	MD	Occipital	Intra	0	-	-	-	-
LBC1936	MD	Occipital	Inter	1	-0.162	-0.162	-0.162	-0.162
LBC1936	MD	Subcortical	Intra	1	-0.159	-0.159	-0.159	-0.159
LBC1936	MD	Subcortical	Inter	2	-0.009	-0.009	-0.180	0.162
Meta-analysis	SC	Frontal	Intra	38	0.044	0.043	0.019	0.104
Meta-analysis	SC	Frontal	Inter	112	0.048	0.043	0.019	0.097
Meta-analysis	SC	Cingulate	Intra	4	0.056	0.046	0.029	0.103
Meta-analysis	SC	Cingulate	Inter	27	0.024	0.027	-0.071	0.102
Meta-analysis	SC	Insula	Intra	0	-	-	-	-
Meta-analysis	SC	Insula	Inter	14	0.035	0.036	-0.029	0.086
Meta-analysis	SC	Temporal	Intra	19	0.047	0.046	-0.027	0.088
Meta-analysis	SC	Temporal	Inter	113	0.045	0.045	-0.029	0.095
Meta-analysis	SC	Parietal	Intra	21	0.054	0.051	0.022	0.099
Meta-analysis	SC	Parietal	Inter	130	0.051	0.049	-0.031	0.102
Meta-analysis	SC	Occipital	Intra	0	-	-	-	-
Meta-analysis	SC	Occipital	Inter	36	0.047	0.044	0.019	0.097
Meta-analysis	SC	Subcortical	Intra	21	0.034	0.029	-0.050	0.109
Meta-analysis	SC	Subcortical	Inter	144	0.047	0.049	-0.071	0.097
Meta-analysis	FA	Frontal	Intra	56	0.055	0.056	0.020	0.111
Meta-analysis	FA	Frontal	Inter	126	0.060	0.060	0.020	0.117
Meta-analysis	FA	Cingulate	Intra	4	0.028	0.027	0.019	0.038
Meta-analysis	FA	Cingulate	Inter	27	0.041	0.039	0.020	0.113
Meta-analysis	FA	Insula	Intra	0	-	-	-	-
Meta-analysis	FA	Insula	Inter	28	0.036	0.037	-0.033	0.077
Meta-analysis	FA	Temporal	Intra	24	0.041	0.039	-0.019	0.086
Meta-analysis	FA	Temporal	Inter	105	0.050	0.049	0.018	0.101
Meta-analysis	FA	Parietal	Intra	23	0.055	0.054	0.022	0.087
Meta-analysis	FA	Parietal	Inter	137	0.058	0.058	0.019	0.107
Meta-analysis	FA	Occipital	Intra	9	0.049	0.044	0.029	0.082
Meta-analysis	FA	Occipital	Inter	52	0.060	0.054	0.019	0.134
Meta-analysis	FA	Subcortical	Intra	13	0.029	0.029	-0.027	0.069
Meta-analysis	FA	Subcortical	Inter	133	0.054	0.053	-0.033	0.134
Meta-analysis	MD	Frontal	Intra	41	-0.069	-0.071	-0.135	-0.020
Meta-analysis	MD	Frontal	Inter	122	-0.074	-0.069	-0.146	-0.030
Meta-analysis	MD	Cingulate	Intra	5	-0.039	-0.035	-0.062	-0.021
Meta-analysis	MD	Cingulate	Inter	40	-0.063	-0.054	-0.139	-0.024
Meta-analysis	MD	Insula	Intra	0	-	-	-	-
Meta-analysis	MD	Insula	Inter	27	-0.052	-0.047	-0.105	-0.021
Meta-analysis	MD	Temporal	Intra	9	-0.027	-0.027	-0.038	-0.019
Meta-analysis	MD	Temporal	Inter	62	-0.043	-0.039	-0.076	-0.020
Meta-analysis	MD	Parietal	Intra	18	-0.067	-0.068	-0.110	-0.032
Meta-analysis	MD	Parietal	Inter	97	-0.065	-0.064	-0.139	-0.020
Meta-analysis	MD	Occipital	Intra	1	-0.020	-0.020	-0.020	-0.020
Meta-analysis	MD	Occipital	Inter	8	-0.032	-0.026	-0.077	-0.020
Meta-analysis	MD	Subcortical	Intra	16	-0.043	-0.034	-0.112	0.021
Meta-analysis	MD	Subcortical	Inter	114	-0.070	-0.072	-0.146	-0.020

Supplementary Table 17. Correlations (Pearson's r) between the meta-analytic node-*g* β coefficients (derived from SC, FA, and MD networks) and cortical morphometry-*g* β coefficients (volume, surface area, and thickness) across 68 cortical regions from the Desikan-Killiany atlas. Bold indicates significance ($p < 0.05$, uncorrected)

	**Volume**		**Surface area**		**Thickness**	
**Weighting**	***r***	***p***	***r***	***p***	***r***	***p***
SC	**0.653**	**0.000**	**0.556**	**0.000**	**0.331**	**0.006**
FA	**0.428**	**0.000**	**0.346**	**0.004**	**0.267**	**0.028**
MD	**-0.406**	**0.001**	**-0.386**	**0.001**	-0.115	0.350

Supplementary Table 18. Moderation estimates for age (standardised estimates) in relation to *g* and global network metrics for the two cohorts with broad age ranges (UKB and STRADL), across weightings (SC, FA, and MD), and across network metrics (mean edge weight, global efficiency, mean clustering coefficient). SE and p-values were estimated by SEM, and q-values were adjusted by FDR.

**Study**	**Weighting**	**Metric**	**β**	***SE***	***p***	***q***
UKB	SC	Mean edge weight	0.000	0.007	0.961	0.961
UKB	SC	Efficiency	0.011	0.007	0.105	0.135
UKB	SC	Clustering	0.006	0.007	0.364	0.409
UKB	FA	Mean edge weight	0.017	0.007	0.018	0.033
UKB	FA	Efficiency	0.013	0.007	0.065	0.097
UKB	FA	Clustering	0.017	0.007	0.017	0.033
UKB	MD	Mean edge weight	-0.042	0.007	0.000	0.000
UKB	MD	Efficiency	-0.047	0.007	0.000	0.000
UKB	MD	Clustering	-0.041	0.007	0.000	0.000
STRADL	SC	Mean edge weight	-0.005	0.033	0.869	0.874
STRADL	SC	Efficiency	0.005	0.033	0.874	0.874
STRADL	SC	Clustering	0.010	0.033	0.752	0.874
STRADL	FA	Mean edge weight	0.039	0.029	0.168	0.555
STRADL	FA	Efficiency	0.037	0.028	0.185	0.555
STRADL	FA	Clustering	0.039	0.028	0.160	0.555
STRADL	MD	Mean edge weight	-0.024	0.030	0.418	0.665
STRADL	MD	Efficiency	-0.025	0.030	0.408	0.665
STRADL	MD	Clustering	-0.023	0.030	0.443	0.665

Supplementary Table 19. Moderation estimates for age (standardised estimates) in relation to *g* and local efficiency for the two cohorts with broad age ranges (UKB and STRADL), for 85 FreeSurfer regions in uncorrected SC networks. *SE* and *p*-values were estimated by SEM, and *q*-values were adjusted by FDR.

		UKB				STRADL			
**Hemisphere**	**Node**	**β**	***SE***	***p***	***q***	**β**	***SE***	***p***	***q***
Left	Thalamus	0.011	0.007	0.117	0.268	-0.034	0.034	0.322	0.830
Left	Caudate	-0.006	0.007	0.350	0.518	0.027	0.029	0.356	0.836
Left	Putamen	0.009	0.007	0.203	0.373	0.031	0.033	0.344	0.836
Left	Pallidum	-0.021	0.007	0.004	0.018	-0.045	0.034	0.182	0.768
Left	Hippocampus	0.035	0.007	0.000	0.000	0.035	0.033	0.291	0.820
Left	Amygdala	0.021	0.007	0.003	0.015	0.091	0.033	0.005	0.183
Left	Accumbens-area	-0.010	0.007	0.167	0.331	0.025	0.031	0.416	0.852
Left	Ventral diencephalon	0.004	0.007	0.532	0.675	0.002	0.036	0.966	0.991
Left	Banks of superior temporal	-0.003	0.007	0.683	0.792	0.039	0.031	0.203	0.768
Left	Caudal anterior cingulate	0.002	0.007	0.739	0.827	-0.028	0.033	0.395	0.845
Left	Caudal middle frontal	-0.021	0.007	0.003	0.015	-0.029	0.031	0.364	0.836
Left	Cuneus	-0.002	0.007	0.783	0.854	0.063	0.032	0.048	0.342
Left	Entorhinal	0.023	0.007	0.001	0.009	0.081	0.030	0.007	0.183
Left	Fusiform	0.025	0.007	0.000	0.004	0.076	0.031	0.013	0.183
Left	Inferior parietal	0.008	0.007	0.236	0.410	-0.003	0.033	0.930	0.991
Left	Inferior temporal	-0.001	0.007	0.876	0.919	-0.004	0.034	0.897	0.991
Left	Isthmus cingulate	-0.027	0.007	0.000	0.002	-0.034	0.035	0.334	0.834
Left	Lateral occipital	0.024	0.007	0.001	0.006	-0.003	0.034	0.939	0.991
Left	Lateral orbitofrontal	0.008	0.007	0.252	0.428	0.019	0.033	0.578	0.927
Left	Lingual	0.004	0.007	0.625	0.759	0.034	0.032	0.292	0.820
Left	Medial orbitofrontal	0.007	0.007	0.342	0.518	0.029	0.029	0.309	0.820
Left	Middle temporal	0.022	0.007	0.002	0.012	-0.022	0.034	0.514	0.891
Left	Parahippocampal	0.037	0.007	0.000	0.000	0.076	0.031	0.012	0.183
Left	Paracentral	0.012	0.007	0.096	0.232	-0.020	0.032	0.541	0.909
Left	Pars opercularis	0.008	0.007	0.275	0.448	-0.026	0.033	0.434	0.858
Left	Pars orbitalis	-0.001	0.007	0.906	0.927	-0.021	0.031	0.491	0.870
Left	Pars triangularis	0.013	0.007	0.062	0.165	-0.001	0.033	0.981	0.991
Left	Pericalcarine	-0.012	0.007	0.094	0.232	0.007	0.031	0.814	0.991
Left	Postcentral	-0.005	0.007	0.479	0.635	0.002	0.033	0.955	0.991
Left	Posterior cingulate	0.003	0.007	0.651	0.777	-0.074	0.035	0.035	0.274
Left	Precentral	-0.000	0.007	0.984	0.984	-0.013	0.035	0.717	0.979
Left	Precuneus	0.014	0.007	0.054	0.153	-0.015	0.032	0.639	0.964
Left	Rostral anterior cingulate	-0.020	0.007	0.006	0.026	-0.009	0.031	0.782	0.991
Left	Rostral middle frontal	0.008	0.007	0.235	0.410	0.019	0.032	0.553	0.909
Left	Superior frontal	0.005	0.007	0.522	0.673	-0.050	0.034	0.144	0.767
Left	Superior parietal	-0.006	0.007	0.422	0.579	0.007	0.035	0.851	0.991
Left	Superior temporal	0.002	0.007	0.799	0.860	-0.030	0.035	0.397	0.845
Left	Supramarginal	-0.009	0.007	0.206	0.373	-0.053	0.034	0.120	0.730
Left	Frontal pole	0.006	0.007	0.365	0.521	-0.005	0.034	0.886	0.991
Left	Temporal pole	0.002	0.007	0.736	0.827	0.024	0.033	0.471	0.859
Left	Transverse temporal	0.005	0.007	0.475	0.635	-0.017	0.033	0.599	0.942
Left	Insula	-0.013	0.007	0.068	0.176	-0.073	0.033	0.028	0.274
Right	Thalamus	0.019	0.007	0.007	0.031	0.019	0.033	0.556	0.909
Right	Caudate	0.039	0.007	0.000	0.000	0.033	0.027	0.217	0.768
Right	Putamen	-0.003	0.007	0.690	0.792	-0.047	0.034	0.162	0.768
Right	Pallidum	0.007	0.007	0.339	0.518	0.002	0.033	0.945	0.991
Right	Hippocampus	0.028	0.007	0.000	0.001	0.035	0.034	0.307	0.820
Right	Amygdala	-0.007	0.007	0.326	0.514	-0.025	0.035	0.466	0.859
Right	Accumbens-area	0.014	0.007	0.053	0.153	-0.035	0.029	0.234	0.797
Right	Ventral diencephalon	0.008	0.007	0.268	0.447	-0.013	0.032	0.691	0.979
Right	Banks of superior temporal	-0.018	0.007	0.010	0.039	-0.070	0.032	0.028	0.274

Right	Caudal anterior cingulate	0.011	0.007	0.120	0.268	-0.001	0.034	0.983	0.991
Right	Caudal middle frontal	-0.019	0.007	0.006	0.027	0.027	0.033	0.421	0.852
Right	Cuneus	-0.015	0.007	0.039	0.127	-0.034	0.033	0.307	0.820
Right	Entorhinal	0.025	0.007	0.000	0.004	0.116	0.037	0.002	0.140
Right	Fusiform	0.008	0.007	0.279	0.448	0.089	0.035	0.012	0.183
Right	Inferior parietal	0.016	0.007	0.023	0.080	0.008	0.031	0.800	0.991
Right	Inferior temporal	0.006	0.007	0.375	0.523	0.001	0.033	0.978	0.991
Right	Isthmus cingulate	-0.025	0.007	0.000	0.004	-0.075	0.035	0.034	0.274
Right	Lateral occipital	0.010	0.007	0.146	0.303	0.070	0.032	0.030	0.274
Right	Lateral orbitofrontal	0.019	0.007	0.008	0.033	0.029	0.034	0.397	0.845
Right	Lingual	-0.006	0.007	0.368	0.521	0.014	0.032	0.658	0.964
Right	Medial orbitofrontal	0.010	0.007	0.172	0.331	-0.033	0.031	0.292	0.820
Right	Middle temporal	0.003	0.007	0.658	0.777	0.011	0.032	0.730	0.979
Right	Parahippocampal	0.029	0.007	0.000	0.001	0.041	0.032	0.208	0.768
Right	Paracentral	0.018	0.007	0.011	0.042	-0.042	0.033	0.207	0.768
Right	Pars opercularis	0.017	0.007	0.018	0.062	-0.009	0.035	0.805	0.991
Right	Pars orbitalis	0.021	0.007	0.003	0.016	0.001	0.032	0.966	0.991
Right	Pars triangularis	-0.000	0.007	0.968	0.980	-0.007	0.033	0.841	0.991
Right	Pericalcarine	-0.001	0.007	0.893	0.925	0.015	0.035	0.675	0.973
Right	Postcentral	-0.004	0.007	0.611	0.753	0.005	0.033	0.874	0.991
Right	Posterior cingulate	0.011	0.007	0.123	0.268	-0.043	0.034	0.213	0.768
Right	Precentral	0.013	0.007	0.059	0.163	-0.015	0.034	0.654	0.964
Right	Precuneus	0.021	0.007	0.003	0.015	-0.000	0.033	0.991	0.991
Right	Rostral anterior cingulate	-0.011	0.007	0.121	0.268	-0.055	0.032	0.084	0.548
Right	Rostral middle frontal	0.014	0.007	0.047	0.143	-0.025	0.033	0.453	0.859
Right	Superior frontal	0.002	0.007	0.749	0.827	-0.038	0.033	0.250	0.816
Right	Superior parietal	0.010	0.007	0.141	0.301	0.049	0.033	0.144	0.767
Right	Superior temporal	-0.001	0.007	0.856	0.909	-0.003	0.032	0.923	0.991
Right	Supramarginal	-0.004	0.007	0.579	0.724	-0.011	0.033	0.737	0.979
Right	Frontal pole	0.010	0.007	0.160	0.325	0.009	0.028	0.764	0.991
Right	Temporal pole	0.010	0.007	0.175	0.331	0.013	0.036	0.712	0.979
Right	Transverse temporal	-0.006	0.007	0.353	0.518	0.024	0.033	0.475	0.859
Right	Insula	-0.014	0.007	0.047	0.143	-0.044	0.032	0.164	0.768
	Brainstem	-0.005	0.007	0.485	0.635	-0.016	0.033	0.632	0.964

Supplementary Table 20. Moderation estimates for age (standardised estimates) in relation to *g* and local efficiency for the two cohorts with broad age ranges (UKB and STRADL), for 85 FreeSurfer regions in FA networks. *SE* and *p*-values were estimated by SEM, and *q*-values were adjusted by FDR.

		UKB				STRADL			
**Hemisphere**	**Node**	**β**	***SE***	***p***	***q***	**β**	***SE***	***p***	***q***
Left	Thalamus	-0.002	0.007	0.820	0.917	0.038	0.029	0.194	0.417
Left	Caudate	0.021	0.007	0.003	0.009	0.052	0.027	0.061	0.417
Left	Putamen	0.001	0.007	0.940	0.957	0.047	0.028	0.097	0.417
Left	Pallidum	-0.013	0.007	0.063	0.122	0.055	0.030	0.065	0.417
Left	Hippocampus	0.007	0.007	0.299	0.423	0.028	0.028	0.327	0.455
Left	Amygdala	0.000	0.007	0.977	0.977	0.044	0.030	0.140	0.417
Left	Accumbens-area	-0.010	0.007	0.169	0.256	0.043	0.028	0.133	0.417
Left	Ventral diencephalon	-0.008	0.007	0.240	0.351	0.030	0.030	0.313	0.455
Left	Banks of superior temporal	-0.005	0.007	0.499	0.642	0.045	0.030	0.125	0.417
Left	Caudal anterior cingulate	0.010	0.007	0.143	0.230	0.029	0.031	0.350	0.455
Left	Caudal middle frontal	0.010	0.007	0.162	0.251	0.031	0.030	0.300	0.455
Left	Cuneus	-0.001	0.007	0.875	0.937	0.003	0.032	0.915	0.926
Left	Entorhinal	0.023	0.007	0.001	0.004	0.014	0.028	0.623	0.671
Left	Fusiform	0.012	0.007	0.088	0.166	0.044	0.027	0.105	0.417
Left	Inferior parietal	0.011	0.007	0.142	0.230	0.033	0.028	0.242	0.417
Left	Inferior temporal	0.001	0.007	0.903	0.939	0.038	0.029	0.186	0.417
Left	Isthmus cingulate	-0.003	0.007	0.709	0.825	0.037	0.029	0.193	0.417
Left	Lateral occipital	0.018	0.007	0.010	0.024	0.027	0.028	0.334	0.455
Left	Lateral orbitofrontal	0.018	0.007	0.010	0.024	0.051	0.029	0.077	0.417
Left	Lingual	0.002	0.007	0.741	0.852	0.030	0.028	0.289	0.455
Left	Medial orbitofrontal	0.023	0.007	0.001	0.004	0.037	0.027	0.176	0.417
Left	Middle temporal	0.005	0.007	0.510	0.647	0.045	0.029	0.116	0.417
Left	Parahippocampal	0.006	0.007	0.428	0.587	0.027	0.031	0.371	0.455
Left	Paracentral	0.011	0.007	0.132	0.221	0.064	0.030	0.036	0.417
Left	Pars opercularis	0.014	0.007	0.047	0.096	0.038	0.029	0.190	0.417
Left	Pars orbitalis	0.039	0.007	0.000	0.000	0.049	0.027	0.072	0.417
Left	Pars triangularis	0.025	0.007	0.001	0.003	0.045	0.028	0.110	0.417
Left	Pericalcarine	0.003	0.007	0.703	0.825	0.036	0.031	0.244	0.417
Left	Postcentral	0.002	0.007	0.807	0.914	0.053	0.030	0.078	0.417
Left	Posterior cingulate	0.019	0.007	0.007	0.019	0.041	0.028	0.147	0.417
Left	Precentral	0.005	0.007	0.447	0.594	0.041	0.030	0.165	0.417
Left	Precuneus	0.003	0.007	0.650	0.778	0.036	0.029	0.210	0.417
Left	Rostral anterior cingulate	-0.000	0.007	0.945	0.957	0.017	0.031	0.594	0.653
Left	Rostral middle frontal	0.030	0.007	0.000	0.000	0.035	0.028	0.217	0.417
Left	Superior frontal	0.029	0.007	0.000	0.000	0.041	0.028	0.145	0.417
Left	Superior parietal	0.005	0.007	0.519	0.649	0.037	0.029	0.206	0.417
Left	Superior temporal	0.001	0.007	0.864	0.937	0.046	0.029	0.111	0.417
Left	Supramarginal	-0.003	0.007	0.634	0.778	0.037	0.029	0.204	0.417
Left	Frontal pole	0.030	0.007	0.000	0.000	0.044	0.029	0.127	0.417
Left	Temporal pole	0.005	0.007	0.444	0.594	0.037	0.030	0.213	0.417
Left	Transverse temporal	-0.013	0.007	0.058	0.116	0.062	0.030	0.040	0.417
Left	Insula	0.001	0.007	0.906	0.939	0.041	0.029	0.160	0.417
Right	Thalamus	0.010	0.007	0.172	0.256	0.030	0.029	0.316	0.455
Right	Caudate	0.021	0.007	0.003	0.010	0.026	0.028	0.351	0.455
Right	Putamen	0.014	0.007	0.047	0.096	0.032	0.029	0.267	0.445
Right	Pallidum	0.011	0.007	0.109	0.194	0.069	0.031	0.025	0.417
Right	Hippocampus	0.021	0.007	0.003	0.010	0.013	0.029	0.654	0.686
Right	Amygdala	0.011	0.007	0.129	0.220	0.000	0.032	0.997	0.997
Right	Accumbens-area	-0.008	0.007	0.262	0.377	0.033	0.033	0.313	0.455
Right	Ventral diencephalon	0.001	0.007	0.852	0.937	0.023	0.030	0.445	0.504
Right	Banks of superior temporal	0.005	0.007	0.497	0.642	0.035	0.029	0.235	0.417
Right	Caudal anterior cingulate	0.020	0.007	0.005	0.016	0.015	0.029	0.600	0.653

Right	Caudal middle frontal	0.030	0.007	0.000	0.000	0.026	0.032	0.419	0.488
Right	Cuneus	-0.006	0.007	0.371	0.517	-0.027	0.031	0.397	0.476
Right	Entorhinal	0.035	0.007	0.000	0.000	0.038	0.030	0.203	0.417
Right	Fusiform	0.025	0.007	0.000	0.002	0.056	0.029	0.053	0.417
Right	Inferior parietal	0.024	0.007	0.001	0.003	0.035	0.029	0.236	0.417
Right	Inferior temporal	0.019	0.007	0.008	0.021	0.040	0.029	0.162	0.417
Right	Isthmus cingulate	0.001	0.007	0.882	0.937	0.025	0.028	0.368	0.455
Right	Lateral occipital	0.026	0.007	0.000	0.002	0.039	0.028	0.169	0.417
Right	Lateral orbitofrontal	0.024	0.007	0.001	0.003	0.042	0.030	0.162	0.417
Right	Lingual	0.012	0.007	0.102	0.189	0.029	0.030	0.323	0.455
Right	Medial orbitofrontal	0.025	0.007	0.001	0.003	0.015	0.032	0.633	0.673
Right	Middle temporal	0.020	0.007	0.006	0.018	0.042	0.029	0.143	0.417
Right	Parahippocampal	0.019	0.007	0.008	0.021	0.024	0.029	0.418	0.488
Right	Paracentral	0.023	0.007	0.001	0.004	0.030	0.029	0.304	0.455
Right	Pars opercularis	0.030	0.007	0.000	0.000	0.028	0.030	0.364	0.455
Right	Pars orbitalis	0.036	0.007	0.000	0.000	0.043	0.029	0.139	0.417
Right	Pars triangularis	0.033	0.007	0.000	0.000	0.033	0.030	0.273	0.447
Right	Pericalcarine	0.015	0.007	0.041	0.089	0.021	0.030	0.467	0.522
Right	Postcentral	0.010	0.007	0.154	0.242	0.047	0.032	0.136	0.417
Right	Posterior cingulate	0.025	0.007	0.000	0.003	0.037	0.028	0.189	0.417
Right	Precentral	0.018	0.007	0.012	0.029	0.029	0.031	0.345	0.455
Right	Precuneus	0.017	0.007	0.015	0.034	0.034	0.028	0.230	0.417
Right	Rostral anterior cingulate	0.015	0.007	0.030	0.067	0.027	0.033	0.425	0.488
Right	Rostral middle frontal	0.031	0.007	0.000	0.000	0.026	0.030	0.375	0.455
Right	Superior frontal	0.031	0.007	0.000	0.000	0.034	0.029	0.245	0.417
Right	Superior parietal	0.011	0.007	0.110	0.194	0.029	0.029	0.312	0.455
Right	Superior temporal	0.011	0.007	0.119	0.207	0.043	0.030	0.150	0.417
Right	Supramarginal	0.021	0.007	0.003	0.010	0.027	0.030	0.364	0.455
Right	Frontal pole	0.032	0.007	0.000	0.000	0.040	0.030	0.181	0.417
Right	Temporal pole	0.026	0.007	0.000	0.002	0.037	0.031	0.222	0.417
Right	Transverse temporal	-0.003	0.007	0.644	0.778	-0.006	0.040	0.873	0.905
Right	Insula	0.014	0.007	0.048	0.096	0.042	0.029	0.151	0.417
	Brainstem	0.022	0.007	0.002	0.007	-0.004	0.033	0.908	0.926

Supplementary Table 21. Moderation estimates for age (standardised estimates) in relation to *g* and local efficiency for the two cohorts with broad age ranges (UKB and STRADL), for 85 FreeSurfer regions in MD networks. *SE* and *p*-values were estimated by SEM, and *q*-values were adjusted by FDR.

		UKB				STRADL			
**Hemisphere**	**Node**	**β**	***SE***	***p***	***q***	**β**	***SE***	***p***	***q***
Left	Thalamus	-0.050	0.007	0.000	0.000	-0.017	0.031	0.579	0.722
Left	Caudate	-0.063	0.007	0.000	0.000	-0.018	0.030	0.544	0.722
Left	Putamen	-0.041	0.007	0.000	0.000	-0.021	0.031	0.487	0.722
Left	Pallidum	-0.037	0.007	0.000	0.000	-0.019	0.031	0.547	0.722
Left	Hippocampus	-0.015	0.007	0.038	0.045	-0.044	0.031	0.157	0.722
Left	Amygdala	-0.002	0.007	0.781	0.781	-0.049	0.030	0.106	0.722
Left	Accumbens-area	-0.003	0.007	0.657	0.665	-0.079	0.031	0.011	0.455
Left	Ventral diencephalon	-0.020	0.007	0.005	0.007	-0.044	0.032	0.171	0.722
Left	Banks of superior temporal	-0.024	0.007	0.001	0.001	-0.016	0.031	0.607	0.737
Left	Caudal anterior cingulate	-0.053	0.007	0.000	0.000	-0.015	0.030	0.627	0.740
Left	Caudal middle frontal	-0.047	0.007	0.000	0.000	-0.008	0.032	0.813	0.856
Left	Cuneus	-0.005	0.007	0.511	0.523	-0.030	0.030	0.326	0.722
Left	Entorhinal	0.006	0.007	0.416	0.442	-0.042	0.032	0.190	0.722
Left	Fusiform	-0.006	0.007	0.383	0.412	-0.040	0.031	0.187	0.722
Left	Inferior parietal	-0.028	0.007	0.000	0.000	-0.018	0.031	0.555	0.722
Left	Inferior temporal	-0.026	0.007	0.000	0.000	-0.026	0.031	0.412	0.722
Left	Isthmus cingulate	-0.038	0.007	0.000	0.000	-0.026	0.030	0.390	0.722
Left	Lateral occipital	-0.022	0.007	0.002	0.003	-0.034	0.030	0.259	0.722
Left	Lateral orbitofrontal	-0.029	0.007	0.000	0.000	-0.043	0.030	0.152	0.722
Left	Lingual	0.006	0.007	0.446	0.468	-0.042	0.030	0.171	0.722
Left	Medial orbitofrontal	-0.050	0.007	0.000	0.000	-0.035	0.030	0.243	0.722
Left	Middle temporal	-0.032	0.007	0.000	0.000	-0.024	0.031	0.447	0.722
Left	Parahippocampal	0.017	0.007	0.018	0.022	-0.030	0.032	0.346	0.722
Left	Paracentral	-0.056	0.007	0.000	0.000	-0.009	0.031	0.769	0.838
Left	Pars opercularis	-0.049	0.007	0.000	0.000	-0.001	0.031	0.968	0.968
Left	Pars orbitalis	-0.053	0.007	0.000	0.000	-0.028	0.030	0.352	0.722
Left	Pars triangularis	-0.052	0.007	0.000	0.000	-0.008	0.031	0.789	0.849
Left	Pericalcarine	-0.010	0.007	0.178	0.196	-0.040	0.031	0.197	0.722
Left	Postcentral	-0.045	0.007	0.000	0.000	-0.007	0.031	0.816	0.856
Left	Posterior cingulate	-0.062	0.007	0.000	0.000	-0.023	0.030	0.429	0.722
Left	Precentral	-0.048	0.007	0.000	0.000	-0.003	0.031	0.915	0.936
Left	Precuneus	-0.035	0.007	0.000	0.000	-0.020	0.031	0.508	0.722
Left	Rostral anterior cingulate	-0.057	0.007	0.000	0.000	-0.037	0.031	0.240	0.722
Left	Rostral middle frontal	-0.064	0.007	0.000	0.000	-0.012	0.030	0.700	0.794
Left	Superior frontal	-0.062	0.007	0.000	0.000	-0.015	0.030	0.626	0.740
Left	Superior parietal	-0.034	0.007	0.000	0.000	-0.019	0.031	0.550	0.722
Left	Superior temporal	-0.033	0.007	0.000	0.000	-0.024	0.031	0.435	0.722
Left	Supramarginal	-0.032	0.007	0.000	0.000	-0.013	0.031	0.665	0.763
Left	Frontal pole	-0.062	0.007	0.000	0.000	-0.017	0.030	0.572	0.722
Left	Temporal pole	-0.006	0.007	0.378	0.412	-0.061	0.030	0.045	0.722
Left	Transverse temporal	-0.028	0.007	0.000	0.000	-0.009	0.030	0.760	0.838
Left	Insula	-0.031	0.007	0.000	0.000	-0.022	0.030	0.453	0.722
Right	Thalamus	-0.049	0.007	0.000	0.000	-0.021	0.030	0.483	0.722
Right	Caudate	-0.060	0.007	0.000	0.000	-0.014	0.030	0.640	0.745
Right	Putamen	-0.048	0.007	0.000	0.000	-0.020	0.030	0.512	0.722
Right	Pallidum	-0.046	0.007	0.000	0.000	-0.030	0.031	0.333	0.722
Right	Hippocampus	-0.022	0.007	0.003	0.004	-0.039	0.030	0.197	0.722
Right	Amygdala	-0.017	0.007	0.021	0.026	-0.043	0.032	0.186	0.722
Right	Accumbens-area	-0.026	0.007	0.000	0.000	-0.085	0.031	0.006	0.455
Right	Ventral diencephalon	-0.038	0.007	0.000	0.000	-0.023	0.031	0.444	0.722
Right	Banks of superior temporal	-0.032	0.007	0.000	0.000	-0.020	0.030	0.503	0.722

Right	Caudal anterior cingulate	-0.055	0.007	0.000	0.000	-0.024	0.030	0.432	0.722
Right	Caudal middle frontal	-0.057	0.007	0.000	0.000	-0.010	0.032	0.749	0.838
Right	Cuneus	-0.011	0.007	0.113	0.128	-0.028	0.030	0.340	0.722
Right	Entorhinal	-0.005	0.007	0.468	0.485	-0.066	0.035	0.064	0.722
Right	Fusiform	-0.016	0.007	0.030	0.036	-0.044	0.030	0.141	0.722
Right	Inferior parietal	-0.042	0.007	0.000	0.000	-0.023	0.030	0.443	0.722
Right	Inferior temporal	-0.029	0.007	0.000	0.000	-0.037	0.030	0.228	0.722
Right	Isthmus cingulate	-0.035	0.007	0.000	0.000	-0.027	0.029	0.353	0.722
Right	Lateral occipital	-0.029	0.007	0.000	0.000	-0.033	0.030	0.278	0.722
Right	Lateral orbitofrontal	-0.044	0.007	0.000	0.000	-0.034	0.030	0.259	0.722
Right	Lingual	-0.017	0.007	0.018	0.023	-0.033	0.030	0.265	0.722
Right	Medial orbitofrontal	-0.057	0.007	0.000	0.000	-0.047	0.031	0.130	0.722
Right	Middle temporal	-0.034	0.007	0.000	0.000	-0.036	0.030	0.237	0.722
Right	Parahippocampal	0.010	0.007	0.167	0.187	-0.033	0.030	0.279	0.722
Right	Paracentral	-0.059	0.007	0.000	0.000	-0.018	0.030	0.541	0.722
Right	Pars opercularis	-0.055	0.007	0.000	0.000	-0.018	0.031	0.549	0.722
Right	Pars orbitalis	-0.054	0.007	0.000	0.000	-0.029	0.030	0.331	0.722
Right	Pars triangularis	-0.051	0.007	0.000	0.000	-0.016	0.030	0.586	0.722
Right	Pericalcarine	-0.014	0.007	0.047	0.054	-0.030	0.031	0.330	0.722
Right	Postcentral	-0.047	0.007	0.000	0.000	-0.017	0.030	0.576	0.722
Right	Posterior cingulate	-0.056	0.007	0.000	0.000	-0.024	0.030	0.435	0.722
Right	Precentral	-0.053	0.007	0.000	0.000	-0.006	0.031	0.842	0.873
Right	Precuneus	-0.041	0.007	0.000	0.000	-0.020	0.031	0.509	0.722
Right	Rostral anterior cingulate	-0.071	0.007	0.000	0.000	-0.048	0.030	0.116	0.722
Right	Rostral middle frontal	-0.065	0.007	0.000	0.000	-0.017	0.030	0.570	0.722
Right	Superior frontal	-0.065	0.007	0.000	0.000	-0.019	0.030	0.520	0.722
Right	Superior parietal	-0.040	0.007	0.000	0.000	-0.019	0.031	0.526	0.722
Right	Superior temporal	-0.036	0.007	0.000	0.000	-0.031	0.030	0.311	0.722
Right	Supramarginal	-0.037	0.007	0.000	0.000	-0.018	0.030	0.540	0.722
Right	Frontal pole	-0.060	0.007	0.000	0.000	-0.026	0.030	0.382	0.722
Right	Temporal pole	-0.015	0.007	0.045	0.052	-0.080	0.034	0.019	0.526
Right	Transverse temporal	-0.029	0.007	0.000	0.000	-0.026	0.040	0.517	0.722
Right	Insula	-0.044	0.007	0.000	0.000	-0.035	0.030	0.240	0.722
	Brainstem	-0.027	0.007	0.000	0.000	-0.003	0.035	0.925	0.936

Supplementary Table 22. Correlations (Pearson's r) between predicted composite scores and actual *g* scores in the UKB held-out sample (*n* = 18,642), alongside those in the three samples contributing to the meta-analysis for comparison.

Sample	SC	FA	MD
UKB held-out	0.256	0.130	0.184
UKB main	0.262	0.140	0.192
STRADL	0.264	0.173	0.128
LBC1936	0.187	0.151	0.084

Figures

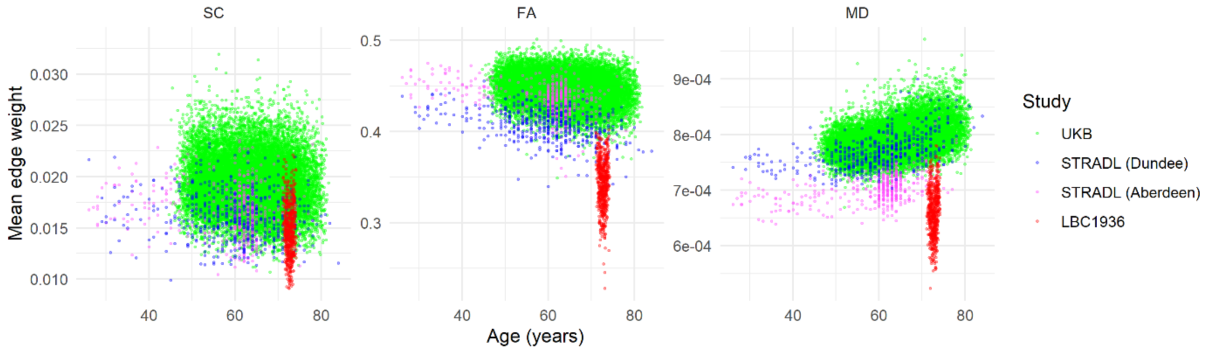

Supplementary Fig. 1. Scatter plots for three network weightings showing mean edge weight against age across the three cohorts, with the STRADL dataset separated into Aberdeen and Dundee scan sites. Network weightings are uncorrected streamline count (SC), fractional anisotropy (FA) and mean diffusivity (MD).

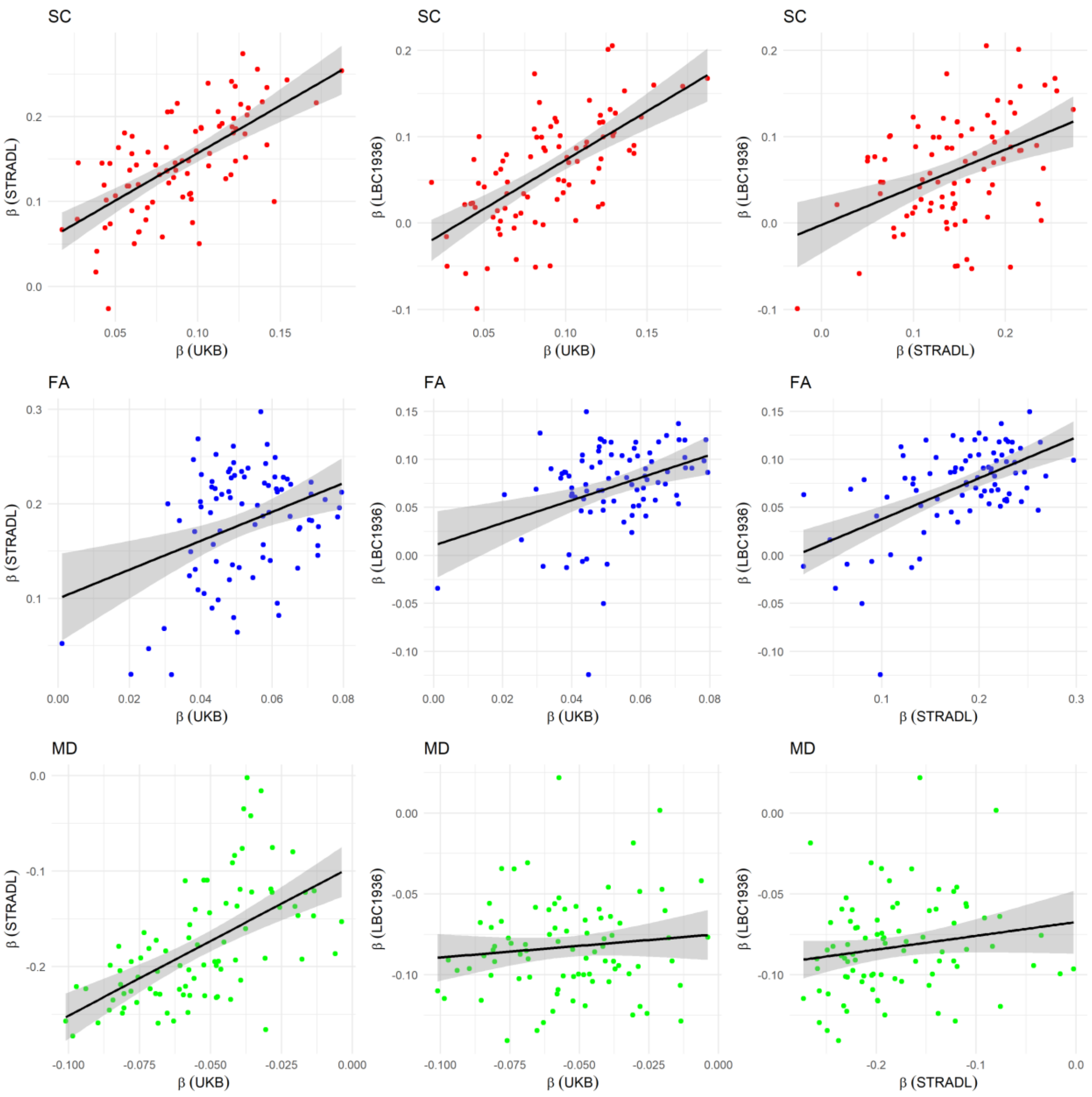

Supplementary Fig. 2. Scatter plots of the relationships between nodal β coefficients for the associations between *g* and nodal local efficiency, for each of the three possible pairings of cohorts across weighting schemes (SC, FA, and MD). Black line indicates linear fit.

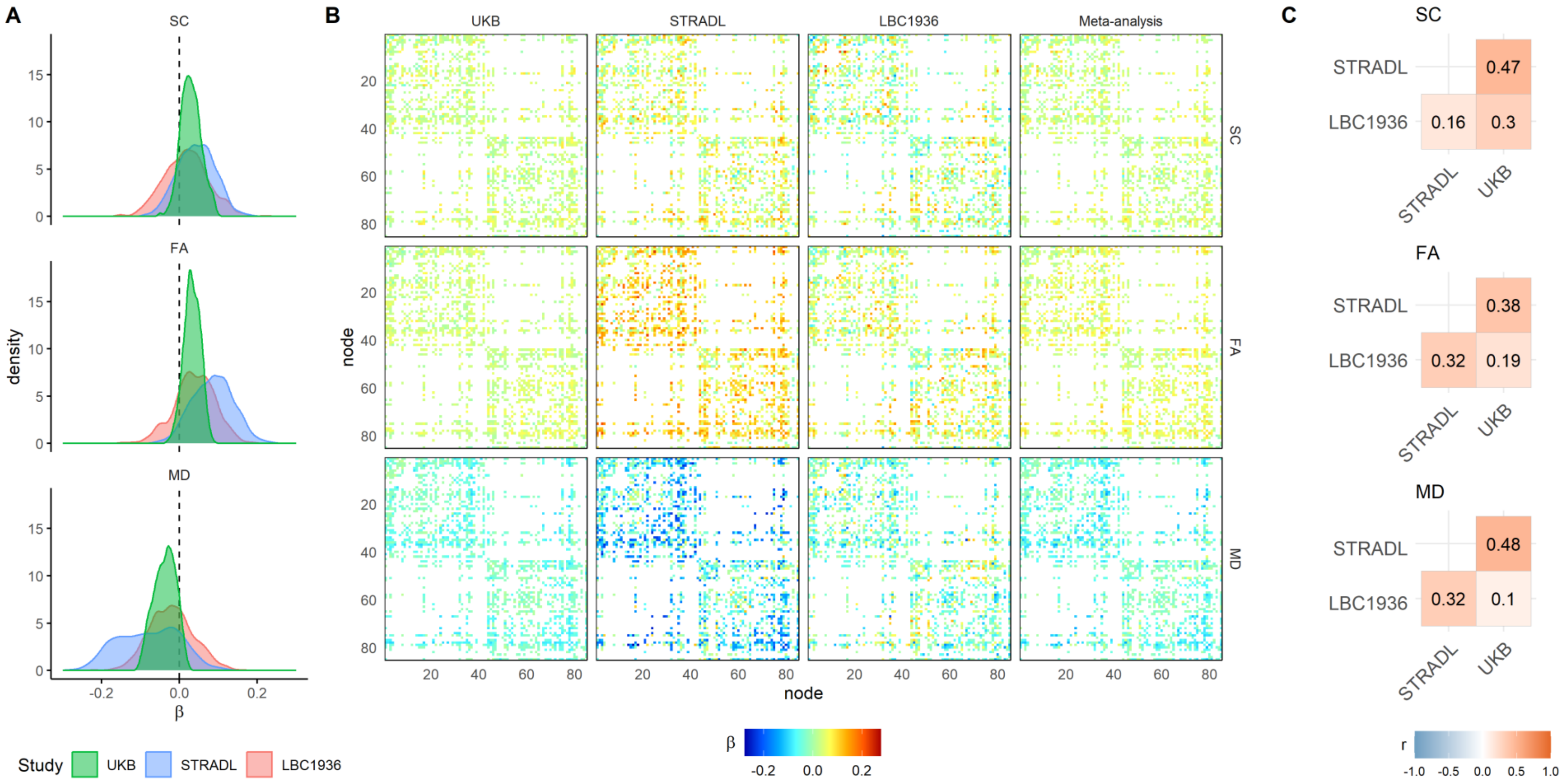

Supplementary Fig. 3. A) Histograms of all regression coefficients (standardised βs) for *g* ~ edge, corrected for age, sex and scanner. B) Connectivity matrices of the estimated βs for each cohort and the meta-analysis, where white indicates an edge was not present in the cross-short network mask. C) Correlations of the above β coefficients between cohorts.

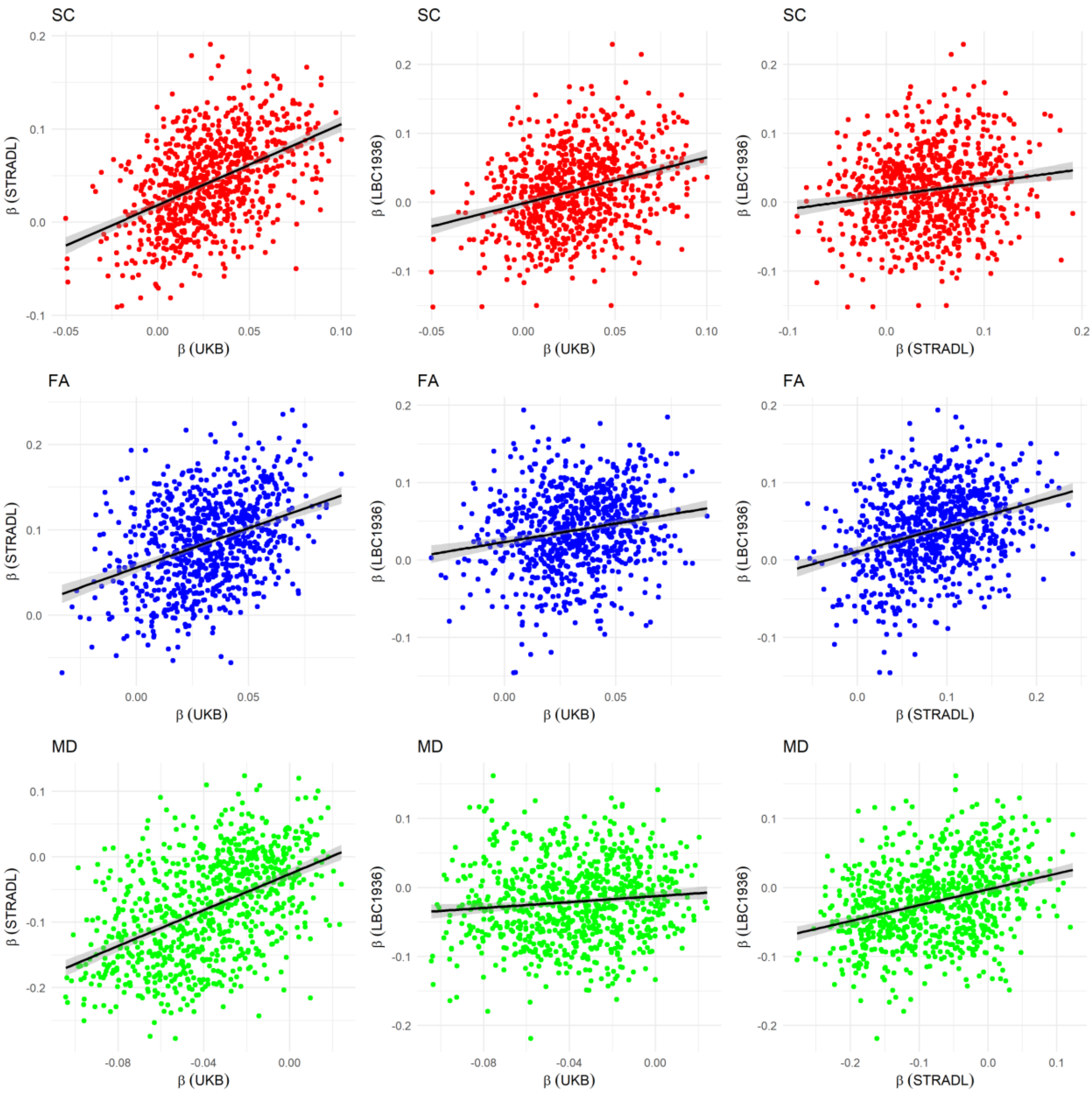

Supplementary Fig. 4. Scatter plots of the relationship between edge-*g* β coefficients for each of the three possible pairings of cohorts across weighting schemes (SC, FA, and MD). Black line indicates linear fit.

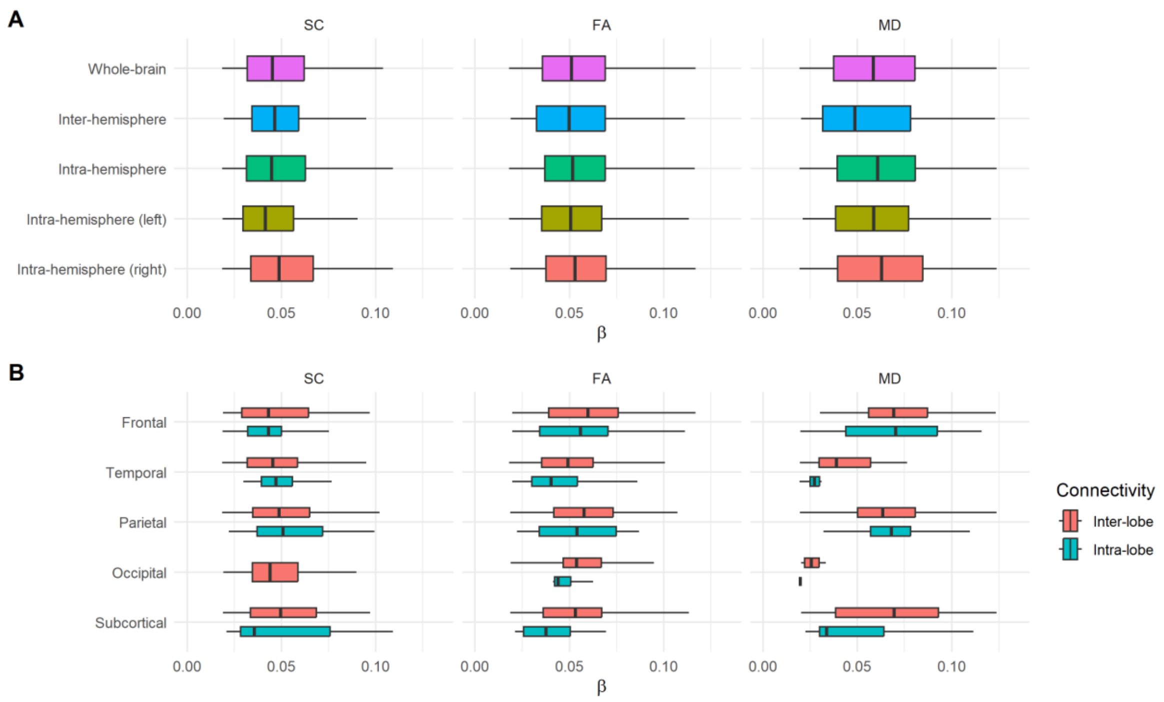

Supplementary Fig. 5. Boxplots of intra- and inter-module connectivity for significant edge-*g* associations ($p < 0.05$, FDR), derived from separate meta-analyses of SC, FA, and MD networks. Modules include: A) Whole-brain (all connections), inter-hemisphere (bilateral), intra-hemisphere (ipsilateral), and intra-hemisphere (left or right only) connections; B) Intra-lobe and inter-lobe (connections to any brain region located outside the designated lobe) for five brain lobes/regions. Insula and cingulate regions were excluded due to an insufficient number of edges. MD βs were sign flipped for plotting.

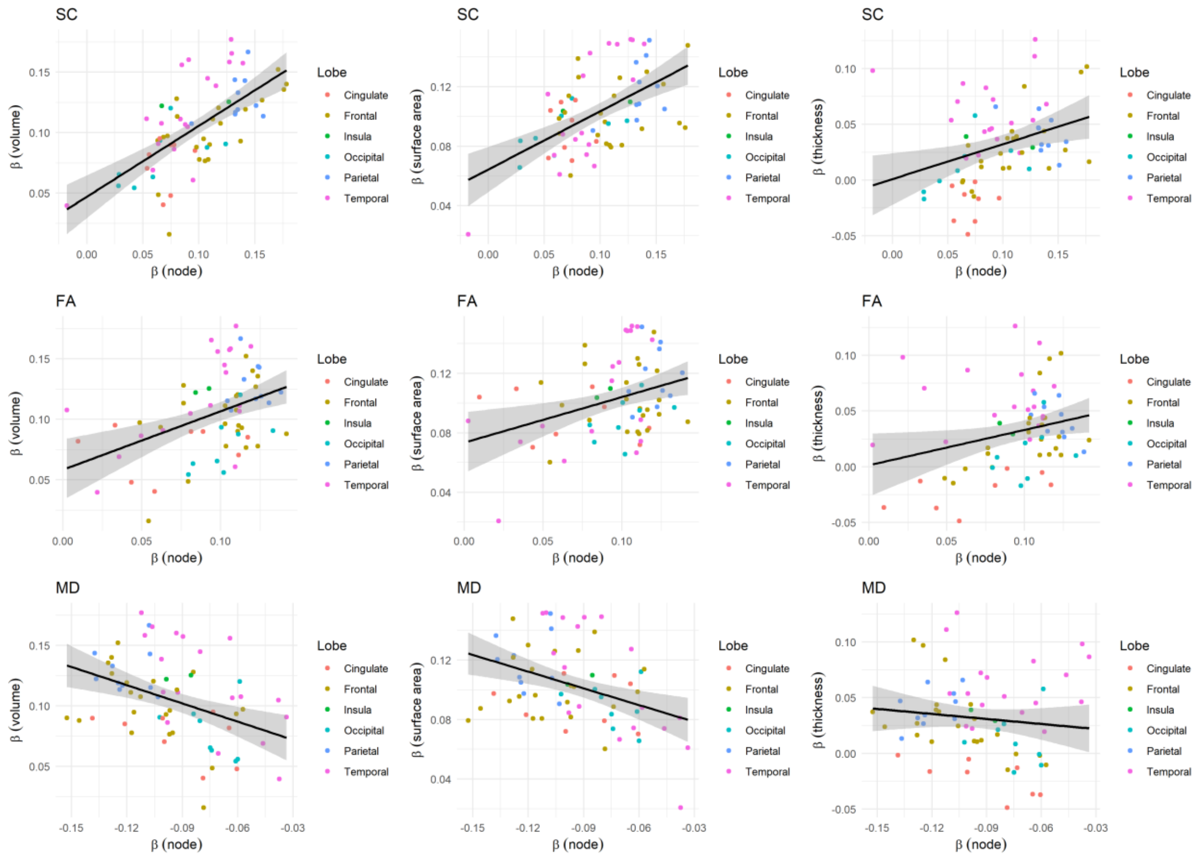

Supplementary Fig. 6: Scatter plots of the relationship between meta-analytic node-*g* β coefficients (derived from SC, FA, and MD networks) and the corresponding cortical morphometry-*g* β coefficients (volume, surface area, and thickness) across 68 cortical regions from the Desikan–Killiany atlas. Points are coloured by lobe, and black lines show linear fits.

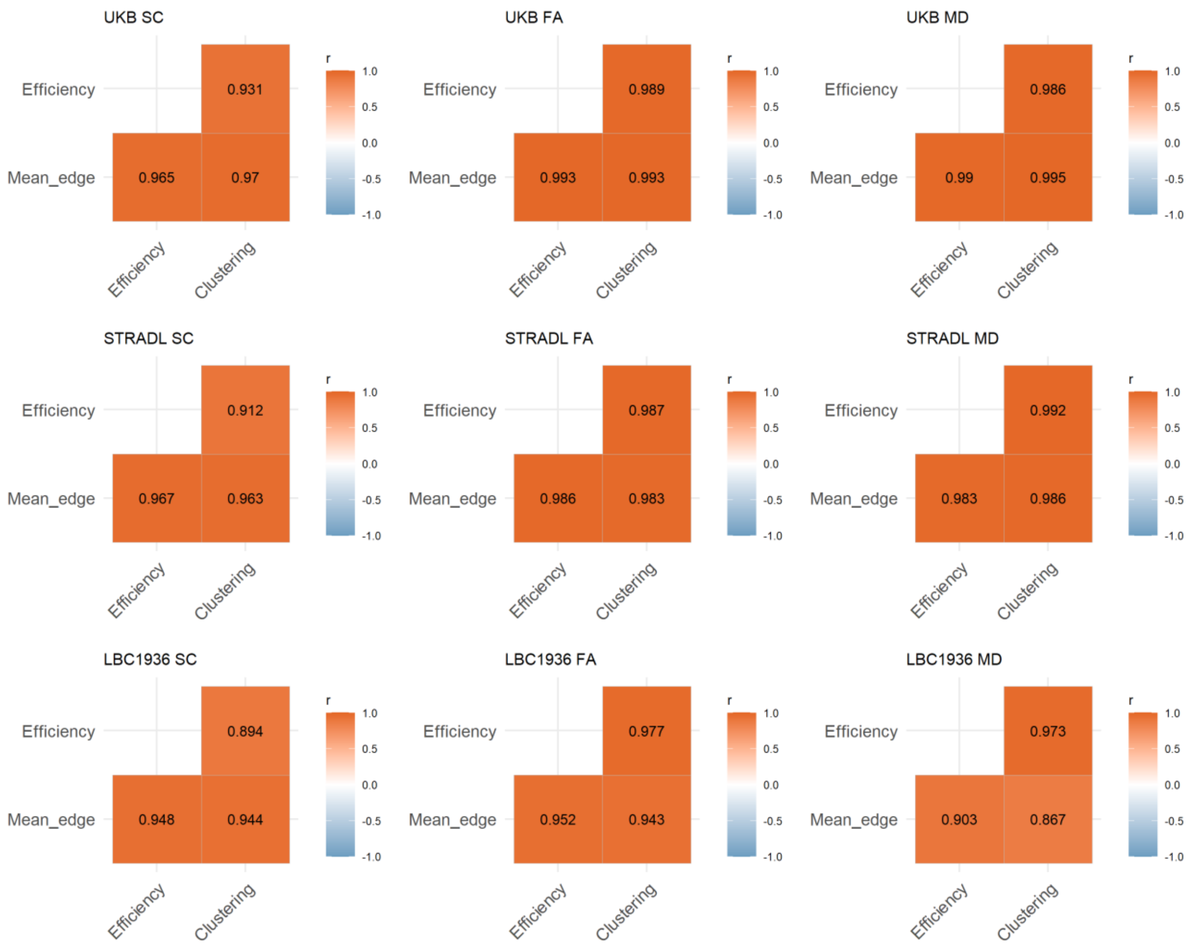

Supplementary Fig. 7. Correlations (Pearson's *r*) among three network metrics (mean edge weight, global efficiency and network clustering coefficient) measured for each cohort and weighting scheme (SC, FA and MD). All correlations were significant ($p < 0.001$, uncorrected).

References

1. Sudlow, C. *et al.* UK Biobank: An Open Access Resource for Identifying the Causes of a Wide Range of Complex Diseases of Middle and Old Age. *PLOS Med.* **12**, e1001779 (2015).
2. Habota, T. *et al.* Cohort profile for the STratifying Resilience and Depression Longitudinally (STRADL) study: A depression-focused investigation of Generation Scotland, using detailed clinical, cognitive, and neuroimaging assessments. *Wellcome Open Res. 2021 4185* **4**, 185 (2021).
3. Deary, I. J., Gow, A. J., Pattie, A. & Starr, J. M. Cohort profile: The lothian birth cohorts of 1921 and 1936. *Int. J. Epidemiol.* **41**, 1576–1584 (2012).
4. Taylor, A. M., Pattie, A. & Deary, I. J. Cohort profile update: The Lothian birth cohorts of 1921 and 1936. *Int. J. Epidemiol.* **47**, 1042-1042r (2018).
5. Miller, K. L. *et al.* Multimodal population brain imaging in the UK Biobank prospective epidemiological study. *Nat. Neurosci.* (2016) doi:10.1038/nn.4393.
6. Smith, B. H. *et al.* Generation Scotland: The Scottish Family Health Study; a new resource for researching genes and heritability. *BMC Med. Genet.* **7**, 1–9 (2006).
7. Deary, I. J. *et al.* The Lothian Birth Cohort 1936: A study to examine influences on cognitive ageing from age 11 to age 70 and beyond. *BMC Geriatr.* (2007) doi:10.1186/1471-2318-7-28.
8. Alfaro-Almagro, F. *et al.* Image processing and Quality Control for the first 10,000 brain imaging datasets from UK Biobank. *Neuroimage* (2018) doi:10.1016/j.neuroimage.2017.10.034.
9. Wardlaw, J. M. *et al.* Brain aging, cognition in youth and old age and vascular disease in the Lothian Birth Cohort 1936: Rationale, design and methodology of the imaging protocol. *Int. J. Stroke* **6**, 547–559 (2011).
10. Desikan, R. S. *et al.* An automated labeling system for subdividing the human cerebral cortex on MRI scans into gyral based regions of interest. *Neuroimage* **31**, 968–980 (2006).
11. Fischl, B. *et al.* Automatically parcellating the human cerebral cortex. *Cereb. Cortex* **14**, 11–22 (2004).
12. Fischl, B. *et al.* Whole brain segmentation: Automated labeling of neuroanatomical structures in the human brain. *Neuron* **33**, 341–355 (2002).
13. Fischl, B. *et al.* Sequence-independent segmentation of magnetic resonance images. *Neuroimage* **23 Suppl 1**, S69-84 (2004).
14. Alfaro-Almagro, F. *et al.* Confound modelling in UK Biobank brain imaging. *Neuroimage* **224**, 117002 (2021).
15. Smith, S. M. *et al.* Advances in functional and structural MR image analysis and implementation as FSL. *Neuroimage* **23**, S208–S219 (2004).
16. Smith, S. M. Fast robust automated brain extraction. *Hum. Brain Mapp.* **17**, 143–155 (2002).
17. Jenkinson, M. & Smith, S. A global optimisation method for robust affine registration of brain images. *Med. Image Anal.* **5**, 143–156 (2001).
18. Pierpaoli, C. & Basser, P. J. Toward a quantitative assessment of diffusion anisotropy. *Magn. Reson. Med.* **36**, 893–906 (1996).
19. Behrens, T. E. J. *et al.* Characterization and propagation of uncertainty in diffusion-weighted MR imaging. *Magn. Reson. Med.* **50**, 1077–1088 (2003).
20. Behrens, T. E. J., Berg, H. J., Jbabdi, S., Rushworth, M. F. S. & Woolrich, M. W. Probabilistic diffusion tractography with multiple fibre orientations: What can we gain? *Neuroimage* **34**, 144–155 (2007).
21. Jbabdi, S., Sotiropoulos, S. N., Savio, A. M., Graña, M. & Behrens, T. E. J. Model-based analysis of multishell diffusion MR data for tractography: How to get over fitting problems. *Magn. Reson. Med.* **68**, 1846–1855 (2012).
22. Buchanan, C. R. *et al.* The effect of network thresholding and weighting on structural brain networks in

the UK Biobank. *Neuroimage* **211**, 116443 (2020).

23. Hagmann, P. *et al.* Mapping the Structural Core of Human Cerebral Cortex. *PLoS Biol.* **6**, 15 (2008).

24. Roberts, J. A., Perry, A., Roberts, G., Mitchell, P. B. & Breakspear, M. Consistency-based thresholding of the human connectome. *Neuroimage* **145**, 118–129 (2017).

25. Rubinov, M. & Sporns, O. Complex network measures of brain connectivity: uses and interpretations. *Neuroimage* **52**, 1059–1069 (2010).

26. Madole, J. W. *et al.* Strong intercorrelations among global graph-theoretic indices of structural connectivity in the human brain. *Neuroimage* **275**, 120160 (2023).

27. Fawns-Ritchie, C. & Deary, I. J. Reliability and validity of the UK Biobank cognitive tests. *PLoS One* **15**, e0231627 (2020).

28. Ritchie, S. J. *et al.* Predictors of ageing-related decline across multiple cognitive functions. *Intelligence* **59**, 115–126 (2016).

29. Johnson, W., Bouchard, T. J., Krueger, R. F., McGue, M. & Gottesman, I. I. Just one g: consistent results from three test batteries. *Intelligence* **32**, 95–107 (2004).

30. Johnson, W., Nijenhuis, J. te & Bouchard, T. J. Still just 1 g: Consistent results from five test batteries. *Intelligence* **36**, 81–95 (2008).

31. Ritchie, K. *et al.* COGNITO: Computerized Assessment of Information Processing. *J. Psychol. Psychother.* **4**, 10 (2014).

32. Smith, A. *Symbol digit modalities test*. (Western Psychological Services, Los Angeles, CA, 1991).

33. Benton, A., Hamsher, K. & Sivan, A. Multilingual Aphasia Examination, 3rd Edition. (1994).

34. Raven, J. C. & Court, J. H. *Raven's progressive matrices and vocabulary scales*. (Oxford Psychologists Press Oxford, 1998).

35. Wechsler, D. Wechsler Adult Intelligence Scale-Third Edition (WAIS-III). (1997).

36. Wechsler, D. Wechsler Adult Intelligence Scale-Third Edition and Wechsler Memory Scale. (1997).

37. Nelson, H. E. & Willison, J. National adult reading test (NART). (1991).

38. Wechsler, D. Wechsler Test of Adult Reading. (2001).

39. Lezak, M. D. *Neuropsychological assessment*. (Oxford University Press, New York, NY, 2004).

40. Deary, I. J. *et al.* The functional anatomy of inspection time: an event-related fMRI study. *Neuroimage* **22**, 1466–1479 (2004).

41. Deary, I. J., Der, G. & Ford, G. Reaction times and intelligence differences: A population-based cohort study. *Intelligence* **29**, 389–399 (2001).

42. Moodie, J. E. *et al.* General and specific patterns of cortical gene expression as substrates of complex cognitive functioning. *bioRxiv* 2023.03.16.532915 (2023) doi:10.1101/2023.03.16.532915.

43. Spearman, C. 'General Intelligence' Objectively Determined and Measured. *Am. J. Psychol.* **15**, 201–293 (1904).

44. Deary, I. J., Penke, L. & Johnson, W. The neuroscience of human intelligence differences. *Nature Reviews Neuroscience* (2010) doi:10.1038/nrn2793.

45. Buchanan, C. R. *et al.* Comparison of structural MRI brain measures between 1.5 and 3 T: Data from the Lothian Birth Cohort 1936. *Hum. Brain Mapp.* **42**, 3905–3921 (2021).

46. Van Den Heuvel, M. P. & Sporns, O. Rich-Club Organization of the Human Connectome. *J. Neurosci.* **31**, 15775–15786 (2011).
